# Deciphering the Transcriptional Regulatory Network Governing Starch and Storage Protein Biosynthesis in Wheat for Breeding Improvement

**DOI:** 10.1002/advs.202401383

**Published:** 2024-06-28

**Authors:** Long Zhao, Jinchao Chen, Zhaoheng Zhang, Wenying Wu, Xuelei Lin, Mingxiang Gao, Yiman Yang, Peng Zhao, Shengbao Xu, Changfeng Yang, Yingyin Yao, Aimin Zhang, Dongcheng Liu, Dongzhi Wang, Jun Xiao

**Affiliations:** ^1^ Key Laboratory of Plant Cell and Chromosome Engineering Institute of Genetics and Developmental Biology Chinese Academy of Sciences Beijing 100101 China; ^2^ College of Advanced Agricultural Sciences University of Chinese Academy of Sciences Beijing 100049 China; ^3^ State Key Laboratory of North China Crop Improvement and Regulation Hebei Agricultural University Baoding Hebei 071001 China; ^4^ State Key Laboratory of Crop Genetics & Germplasm Enhancement and Utilization Nanjing Agricultural University Nanjing Jiangsu 210095 China; ^5^ State Key Laboratory for Crop Stress Resistance and High‐Efficiency Production College of Agronomy Northwest A&F University Yangling 712100 China; ^6^ State Key Laboratory for Agrobiotechnology, Key Laboratory of Crop Heterosis Utilization (MOE) China Agricultural University Beijing 100193 China; ^7^ Centre of Excellence for Plant and Microbial Science (CEPAMS) JIC‐CAS Beijing 100101 China

**Keywords:** epigenetics, seed storage protein, starch, TRN, wheat endosperm

## Abstract

Starch and seed storage protein (SSP) composition profoundly impact wheat grain yield and quality. To unveil regulatory mechanisms governing their biosynthesis, transcriptome, and epigenome profiling is conducted across key endosperm developmental stages, revealing that chromatin accessibility, H3K27ac, and H3K27me3 collectively regulate SSP and starch genes with varying impact. Population transcriptome and phenotype analyses highlight accessible promoter regions’ crucial role as a genetic variation resource, influencing grain yield and quality in a core collection of wheat accessions. Integration of time‐serial RNA‐seq and ATAC‐seq enables the construction of a hierarchical transcriptional regulatory network governing starch and SSP biosynthesis, identifying 42 high‐confidence novel candidates. These candidates exhibit overlap with genetic regions associated with grain size and quality traits, and their functional significance is validated through expression‐phenotype association analysis among wheat accessions and loss‐of‐function mutants. Functional analysis of *wheat abscisic acid insensitive 3‐A1* (*TaABI3‐A1*) with genome editing knock‐out lines demonstrates its role in promoting SSP accumulation while repressing starch biosynthesis through transcriptional regulation. Excellent *TaABI3‐A1^Hap1^* with enhanced grain weight is selected during the breeding process in China, linked to altered expression levels. This study unveils key regulators, advancing understanding of SSP and starch biosynthesis regulation and contributing to breeding enhancement.

## Graphical abstract

Transcriptome and epigenome profiles throughout endosperm developmental stages illuminate epigenetic regulation of starch and seed storage protein expression dynamics, which shapes wheat grain traits within the population. Integrating transcriptional regulatory networks and population genetics unveils novel regulators for improving grain yield and quality in wheat.

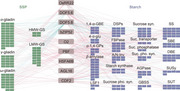

## 1 Introduction

Wheat stands as a vital global staple crop worldwide, contributing 20% of daily calorie intake and 15% of protein consumption. Given the expanding population and evolving climates, breeding initiatives are pivotal for enhancing both yield and quality.^[^ ^1^ ^]^ Endosperm development notably shapes grain yield and quality, initiating post‐cellularization with the outer layer forming the aleurone layer and inner cells becoming starch and protein storage cells.^[^ ^2^ ^]^ During grain filling, essential storage molecules like starch and gluten proteins are synthesized and accumulated.^[^ ^3^ ^]^ Starch constitutes 70% of dry weight in mature seeds, and seed storage protein (SSP) is pivotal for determining wheat flour's end‐use quality and viscoelastic properties to the dough.^[^ ^4^, ^5^ ^]^

The intricate process of wheat grain starch biosynthesis relies on a suite of enzymes and transporters known as starch synthesis‐related genes (SSRGs). These include key players like ADP‐glucose pyrophosphorylases (AGPases), granule‐bound starch synthases (GBSSs), starch synthases (SSs), starch branching enzymes (SBE), debranching enzymes (DBEs), and starch/*α*‐glucan phosphorylases (PHOs), orchestrating various steps of starch synthesis.^[^ ^6^ ^]^ AGPase, serving as the initial key regulatory enzyme, facilitates the conversion of glucose‐1‐phosphate and ATP to ADP‐glucose (ADPG). GBSS then utilizes ADP‐glucose to construct linear glucose residues, thus generating amylose. SS extends glucan chains, while SBE and DBE contribute to the branched structure of amylopectin.^[^ ^7^, ^8^ ^]^ Transcription factors (TFs) also play vital roles in regulating SSRGs, such as Rice Starch Regulator 1 (RSR1),^[^ ^9^ ^]^ Basic leucine‐zipper TF 28 (TabZIP28),^[^ ^10^ ^]^ and the endosperm‐specific NAM/ATAF/CUC (NAC) TF TaNAC019,^[^ ^11^, ^12^ ^]^ and governing seed starch content and grain weight.

Gluten, a vital component in wheat influencing dough properties, comprises glutenins and gliadins.^[^ ^13^ ^]^ Glutenins, including high‐molecular‐weight (HMW) and low‐molecular‐weight (LMW) subunits, form essential gluten polymers for dough elasticity.^[^ ^14^ ^]^ Gliadins, categorized as *α*/*β*‐, *γ*‐, *ω*‐, and *δ*‐gliadins, contributing to the dough viscosity by interacting with glutenins.^[^ ^15^ ^]^ Their transcriptional regulation in grain endosperm development involves intricate networks and TFs such as Storage Protein Activator (SPA), SPA Heterodimerizing Protein (SHP), storage protein repressor (SPR), Prolamin box (P‐box) binding factor (PBF), TaFUSCA3, TaNAC019, TaQM, TaB3‐2A1 and TaGAMyb. These factors interact to modulate gluten gene expression, significantly impacting dough characteristics. SPR and SHP act as a suppressor of SSP synthesis,^[^ ^16^ ^]^ while SPA activates the expression of HMW‐GS and LMW‐GS genes.^[^ ^17^ ^]^ Additionally, PBF, TaQM, and TaB3‐2A1 regulate the LMW‐GS, HMW‐GS, and gliadin genes.^[^ ^18^, ^19^, ^20^, ^21^, ^22^ ^]^ TaGAMyb activates HMW‐GS genes through histone H3 acetylation,^[^ ^23^ ^]^ and TaNAC019 interacts with TaGAMyb to activate glutenin genes.^[^ ^12^ ^]^ TaFUSCA3, a B3‐superfamily TF, also activates the HMW‐GS subunit gene *TaGlu‐1Bx7* through RY repeat binding sites.^[^ ^18^ ^]^

Genetic manipulation of starch and SSP genes has been a cornerstone strategy in wheat breeding. The *Waxy* (*Wx*) gene, pivotal for amylose synthesis, has been extensively harnessed, enabling the development of transgenic lines with varied amylose content (AC) and facilitating the creation of partial waxy and amylose‐free waxy wheat by combining null *wx* alleles.^[^ ^24^, ^25^ ^]^ Induced mutations in *TaSBEII*, *TaSSII*, and *TaSSIIIa* genes have yielded wheat varieties with elevated AC and resistant starch.^[^ ^26^, ^27^, ^28^ ^]^ Additionally, significant improvements in wheat quality have been achieved through targeted manipulation of main SSP genes, such as *Grain protein content‐B1* (*GPC‐B1*) and *HMW‐GS* alleles.^[^ ^29^, ^30^, ^31^ ^]^ Beyond direct modification of starch and SSP genes, manipulating transcriptional regulators like *TaNAC019* and *TaNAC‐A18* has proven invaluable in wheat breeding practices.^[^ ^12^, ^32^ ^]^ To overcome yield limitations and balancing the trade‐off between yield and quality in wheat, there's a pressing need to systematically identify genetic regulators for wheat endosperm development. This involves unraveling the molecular mechanism of SSP and starch synthesis and understanding the intricate interactions and coordination between these vital aspects.

Despite advances in identifying genetic loci for grain size, SSP, and starch‐related traits through QTL analysis and genome‐wide association studies (GWAS), the cloning of these genes has been limited by challenges such as mapping accuracy and understanding causal variants and genes due to linkage disequilibrium.^[^ ^33^, ^34^, ^35^, ^36^, ^37^ ^]^ To overcome these hurdles, a comprehensive approach integrating multi‐layer data, including transcriptome,^[^ ^38^, ^39^ ^]^ translatome,^[^ ^40^ ^]^ metablome,^[^ ^41^ ^]^ and chromatin accessibility,^[^ ^42^ ^]^ has been implemented. This integrated approach aims to identify key regulators in wheat spike and grain development and enhance breeding practices.^[^ ^43^, ^44^ ^]^

In this study, we performed a comprehensive analysis of the transcriptome and epigenome profiles throughout grain development stages using *cv*. Chinese Spring (CS). Our investigation delved into the dynamic transcriptome changes associated with starch and protein biosynthesis, linking variations in expression with the phenotypic diversity across the population. By integrating transcriptional regulatory networks (TRN), GWAS data, gene‐indexed mutant libraries, and population transcriptome information, we identified pivotal factors that demonstrate the potential for synergistic regulation of SSP and starch synthesis.

## 2 Results

### 2.1 Transcriptome and Chromatin Landscapes during Wheat Grain Development

**Figure 1:**
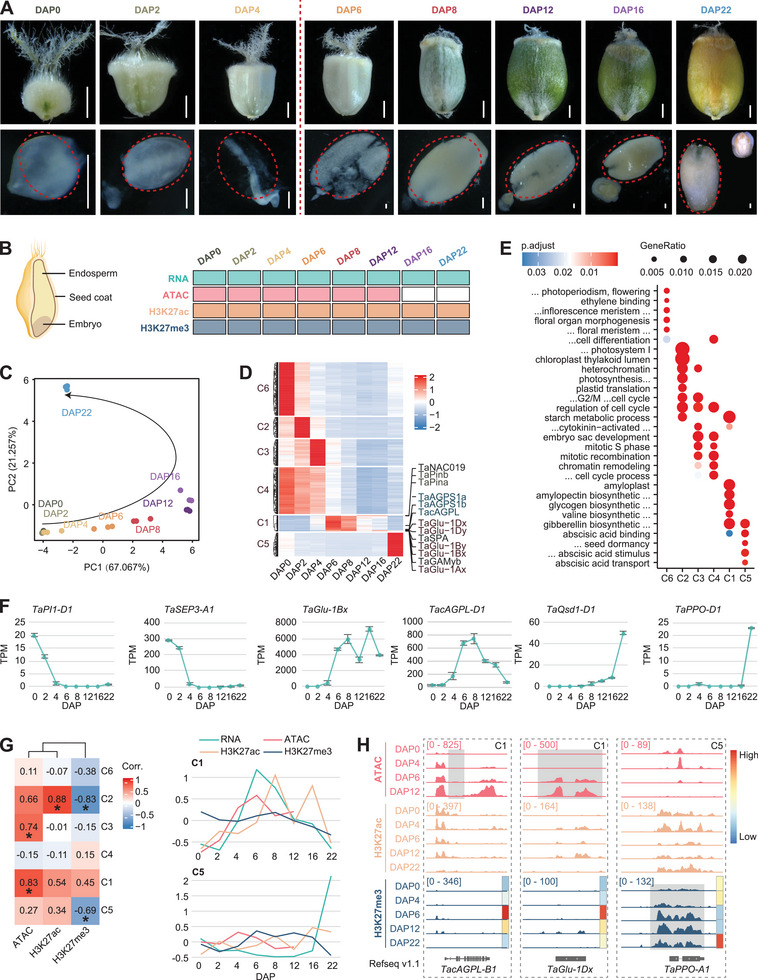
Transcription and epigenetic modification dynamics during grain development in wheat. A) Morphology of developing seeds from 0–22 days after pollination (DAP0‐22) in wheat (upper); the dashed circle indicates tissue samples used for transcriptome and epigenome analysis at indicated DAPs (lower), Bar = 2 cm. B) Diagram indicating the general structure of wheat seed and experimental design of sampling. Endosperm tissues of different DAPs are used for RNA‐seq, ATAC‐seq, and CUT&Tag analysis for H3K27ac, and H3K27me3. C) PCA analysis of time‐serial RNA‐seq data, three bio‐replicates were sequenced at each developing time point. D) K‐mean clustering of differentially expressed genes with grain development, starch, and storage protein‐coding genes, and some known transcription factors are highlighted. E) Gene Ontology enrichment of different clusters in (D). F) Representative genes from different clusters were exhibited with varied dynamic expression patterns during grain development from DAP0‐22. G) Heatmap showing Pearson correlation coefficient between gene expression and epigenetic modifications in different clusters (left), the asterisks indicated the significant correlation with *P* ≤ 0.05. Line plot showing the dynamic of Z‐score normalized average value of gene expression TPM and epigenetic modification peaks CPM at different DAP. H) IGV showing the various epigenetic modification tracks of representative genes *TacAGPL‐B1* and *TaGlu‐1Dx* from cluster1 and *TaPPO‐A1* from cluster5. The color bar on the right side showing the normalized gene expression level.

To explore the molecular mechanisms underlying starch biosynthesis and SSP accumulation during wheat endosperm development, we analyzed the transcriptional dynamics and chromatin landscapes across key developmental stages, spanning from 0–22 days after pollination (DAP0‐22) (**Figure** **1A,B**). Due to technical challenges, we harvested the embryo sac before DAP4, while isolating the endosperm at DAP6 and subsequent stages (Figure 1A,B). Unfortunately, the high starch content in the late stages of endosperm development, coupled with the necessity for high purity of nuclei for Assay for Transposase‐Accessible Chromatin with highthroughput sequencing (ATAC‐seq) compared to Cleavage Under Targets and Tagmentation (CUT&Tag),^[^ ^45^ ^]^ hindered our ability to examine chromatin accessibility beyond DAP12 (Figure 1B).

A total of 60241 genes were found to be expressed (TPM ≥ 0.5) in at least one sample during endosperm development (Table S1, Supporting Information). Principal component analysis (PCA) revealed a progressive developmental trajectory, grouping into three stages that mirror cellular transformations (Figure 1C; Figure S1A, Supporting Information). The global transcriptome exhibited high dynamics, with 96% of genes considered as differentially expressed genes (DEGs) among various samples (Figure S1B, Supporting Information). The number of specifically expressed and generally expressed genes (TPM ≥ 0.5) were lowest between DAP12‐DAP16, followed by an increase at DAP22, which was not due to sequencing bias (Figure 1D; Figure S1C–F, Supporting Information). Most triads among the three sub‐genomes were balanced in expression, highly correlated with epigenetic modification levels (Figure S1C,G,H, Supporting Information). DEGs were categorized into six clusters based on their temporal expression patterns, associated with distinct enrichment of Gene Ontology terms and specific TF families (Figure 1D,E; Figure S1I, Table S2, Supporting Information). Interestingly, genes involved in gluten protein and starch biosynthesis primarily belonged to cluster1 (C1), such as *TacAGPL‐D1* (encoding the cytosolic large subunit) and *TaGlu‐1Bx*, showing high expression levels at DAP6 and DAP8, persisting until DAP16 (Figure 1D–F). In contrast, Cluster6 (C6) genes were predominantly expressed in unfertilized samples and enriched with flower organ identity genes like *TaSEP3‐A1* ^[^ ^46^ ^]^ and *TaPI1‐D1* ^[^ ^47^ ^]^ (Figure 1D–F). Cluster5 (C5) genes were specifically expressed at DAP22 and related to seed dormancy, including *TaQsd1‐D1* ^[^ ^48^ ^]^ and *TaPPO‐A1* ^[^ ^49^ ^]^ (Figure 1D–F).

We proceeded to analyze the dynamics of the chromatin landscape during grain development. PCA of ATAC‐seq data delineated a clear developmental trajectory, although this clarity wasn't mirrored in the H3K27me3 and H3K27ac data (Figure S1J, Supporting Information). Intriguingly, different clusters of temporally expressed genes showed correlated with distinct epigenetic modifications (Figure 1G,H; Figure S1K, Supporting Information). For instance, genes in C5 exhibited a negative correlation with H3K27me3, while genes in C2 displayed a positive correlation with H3K27ac and a negative correlation with H3K27me3 dynamics. Notably, genes in C3 and C1 showed a positive correlation with chromatin accessibility (Figure 1G,H; Figure S1K, Supporting Information). In particular, genes related to starch biosynthesis and gluten accumulation, such as *TacAGPL‐D1* and *TaGlu‐1Dx* within C1, demonstrated increased expression and chromatin accessibility from DAP6 to DAP12 (Figure 1H). Whereas, *TaPPO‐A1* within C5 showed specific activation at DAP22, concurrent with the decline in H3K27me3 levels (Figure 1H).

The dynamic and high expression of various gene types during endosperm development reflects the distinctive character of each stage. The distinct correlation between genes in different clusters and their transcriptional and chromatin features indicates a diverse regulatory pattern for stage‐specific high‐expression genes.

### 2.2 Epigenetic Regulation of Starch and SSP Genes’ Expression Dynamics

**Figure 2:**
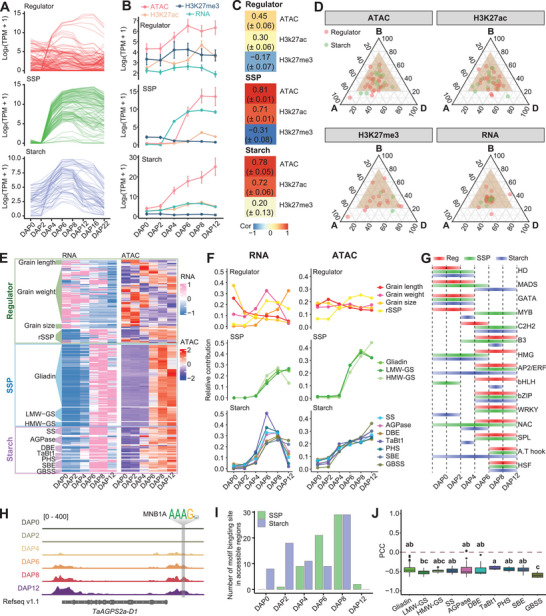
Robust correlation between epigenetic modification and dynamic expression of starch and SSP coding genes. A) The expression patterns of SSP coding, starch synthesis, and regulators genes during DAP0‐22. B) The expression and epigenetic modification levels of SSP, Starch, and Reg‐genes are observed from DAP0‐12. Each dot represents the average value of the gene in each group, and the error bars represent the average value ± standard error (SE). C) Heatmap showing Pearson correlation coefficient (PCC) between genes’ expression and epigenetics modification in three gene groups. The PCC between the expression levels of all genes in each group and the levels of epigenetic modifications is calculated, and then the average ± standard error (SE) of PCC in each group was displayed. D) Ternary plot showing relative expression and epigenetics modification (ATAC, H3K27ac, and H3K27me3) abundance of starch biosynthesis and regulators genes. Each circle represents a gene triad with A, B, and D coordinates consisting of the relative contribution of each homoeolog to the overall triad. Balanced triads are shown within a brown shade. E) Gene expression (left) and chromatin accessibility (right) at proximal accessible regions (pACRs) of three‐group genes and further sub‐classification of each group. F) Expression (left) and chromatin accessibility at pACRs (right) patterns of each subgroup gene during endosperm development. The average TPM expression of sub‐groups (based on Figure 2E) at all stages was calculated. The relative contribution of each sub‐group in each stage was then normalized to the average TPM expression of that sub‐group divided by the total average TPM expression of all stages. G) Diagram showing the TF binding activity of each family across developmental stages based on chromVAR. H) IGV showing the chromatin accessibility and binding motif of Dof family TF at the promoter of *TaAGPS2a‐D1*. The motif and footprint dynamic of Dof TF were shown. I) The number of MNB1A binding motifs within the accessible regions of starch and SSP coding genes during endosperm development. J) The boxplot of PCC between gene expression of each subgroup of starch and SSP coding genes and *MNB1A*. The LSD multiple comparisons test was used to determine the significance of PCC differences among subtype genes. Different letters indicate a significant difference at *P* ≤ 0.05.

Given the significant impact of starch and SSP biosynthesis and composition on grain yield and quality in wheat,^[^ ^4^ ^]^ we conducted a detailed analysis of SSP coding genes (SSP), major starch synthesis genes (Starch), and regulators (Reg), including SSP regulator (rSSP) and grain trait regulators (GL, GW, TGW, and GS indicates genes regulates grain length, grain width, thousand‐grain weight, and grain size, respectively), examining their expression and epigenetic modification patterns during endosperm development (Table S3, Supporting Information).^[^ ^50^ ^]^ SSP genes began expressing at DAP4 and declined post‐DAP20, while Starch genes initiated at DAP4 and decreased earlier after DAP8 (**Figure** **2A**). Reg‐genes exhibited diverse expression patterns, aligning with different grain developmental traits (Figure 2A).^[^ ^51^, ^52^, ^53^ ^]^ We analyzed the correlation between transcriptional dynamics and epigenetic modifications from DAP0 to DAP12 (Figure 2B,C; Figure S2A, Supporting Information), as ATAC‐seq was conducted only until DAP12 (Figure 1B). Transcription levels of Starch genes showed a strong positive correlation with chromatin accessibility (*R* = 0.78) and H3K27ac dynamics (*R* = 0.71), while H3K27me3 dynamics showed a weaker negative correlation (*R* = –0.31) with transcription profiles. Similarly, for SSP genes, chromatin accessibility and H3K27ac exhibited a strong positive correlation (*R* = 0.81 and *R* = 0.71, respectively), while H3K27me3 did not show any correlation (*R* = –0.20) with transcription dynamics. However, the expression of Reg‐genes was not generally associated with epigenetic modifications, possibly due to the diverse expression patterns of different regulators (Figure 2B,C; Figure S2A, Supporting Information). We further analyzed the subgenome expression preferences among triads of Starch and Reg‐genes, opting not to include SSP genes in the analysis due to their ambiguous annotation and uncertain homoeologs. The expression of Starch and Reg‐genes exhibited a primarily balanced pattern, while epigenetic modifications showed greater subgenomic differentiation, indicating an integrated effect of various epigenetic modifications on transcription (Figure 2D; Figure S2B, Supporting Information).

Different subclasses within the three gene sets exhibited relatively similar expression patterns associated with their roles in mediating and regulating starch and SSP biosynthesis (Figure 2E,F). For instance, Reg‐genes displayed two distinct expression patterns, with GL and GS regulators high expressed at early‐stage (DAP0‐4), while rSSP was highly expressed in mid‐stage (DAP6‐12). Chromatin accessibility dynamics aligned well with the expression patterns of these subclasses (Figure 2E,F). Similarly, within Starch genes, *TaBt1* (Brittle1 transporter) expression initiated earliest, while SBE and GBSS genes expression occurred latest, associated with slight variations in chromatin accessibility gains. In contrast, all SSP genes exhibited a similar expression and synchronized chromatin accessibility pattern (Figure 2E,F). In summary, chromatin accessibility showed a high correlation with subcategories of Reg, Starch, and SSP genes’ expression (Figure S2C, Supporting Information).

Giving the strong correlation between chromatin accessibility and transcription dynamics, we employed chromVar to analyze the activity of TF binding motifs in the accessible regions of these genes (Figure 2G). TF activities showed significant differences between early endosperm development (DAP0‐4) and grain‐filling stages (DAP6‐12) (Figure S2D, Supporting Information). C2H2, AP2, and bZIP displayed higher activity in the accessible promoter regions of SSP and Starch genes during grain‐filling stages, suggesting their involvement in regulating grain storage substance accumulation (Figure 2G). For HMW‐GS, key factors in end‐use quality regulation, we identified conserved binding motifs of bZIP, NAC, and MYBs in the promoter region (Figure S2E, Supporting Information). Notably, a gene belonging to the C2H2 TF family, Maize Dof1 (MNB1A),^[^ ^54^, ^55^ ^]^ exhibited strong activity in the accessible region of numerous SSP and starch biosynthesis genes during the DAP6‐DAP12 stage (Figure 2H; Figure S2F, Supporting Information). The number of binding motifs that located in the open chromatin regions increases during endosperm development, peaking at DAP8. (Figure 2I). Furthermore, the expression of *MNB1A*, which decreased and bottomed at DAP6‐8 before gradually increasing, was significantly negatively correlated with SSP and Starch genes (Figure 2J; Figure S2G,H, Supporting Information).

In conclusion, a robust correlation exists between epigenetic modifications and the expression of starch biosynthesis and SSP genes. This correlation is highlighted by the presence of specific cis‐motifs and recognition TFs associated with accessible promoter regions in a timely manner.

### 2.3 Genetic and Expression Variability of Starch and SSP Genes Shapes Wheat Grain Traits

**Figure 3:**
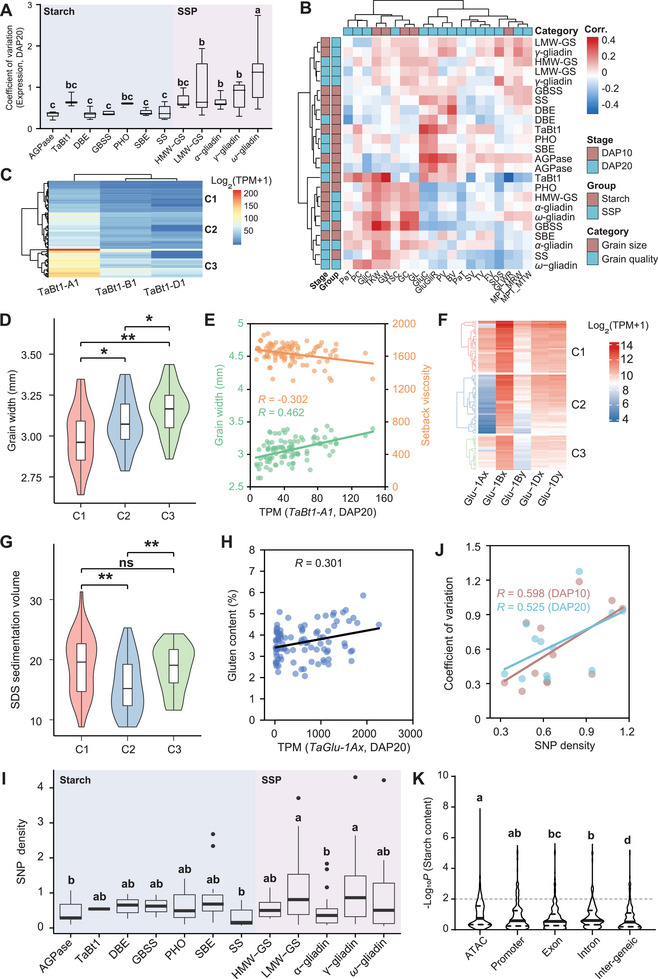
Variation in the regulatory region alters expression and influences grain size and the quality of the wheat population. A) The comparison of the coefficient of variation (CV) in expression levels for each subtype gene within the core collection of 102 wheat accessions at DAP20. The box denotes the 25th, median, and 75th percentiles, and the whiskers indicate the 1.5× interquartile range. The LSD multiple comparisons test was used to determine the significance of CV differences among subtype genes. Different letters indicate a significant difference at *P* ≤ 0.05. B) A heatmap showing the correlation between phenotypic traits and the expression level of subtype genes. C) Expression level of *TaBt1* genes in developing grains at DAP20 within the wheat population. C1, C2, and C3 are clustered by the TPM value with the “complete” method using pheatmap. D) Comparison of the grain width of wheat accessions among three groups. The student's *t*‐test was used to determine the statistical significance between the two groups. *, *P* ≤ 0.05; **, *P* ≤ 0.01. E) Scatter plot of grain width (green dots) and setback viscosity (brown dots) against the expression level values of *TaBt1‐A1* in the corresponding accession at DAP20. Each dot denotes one accession, and the lines represent the regression trend calculated by the general linear model. F) Expression level of HMW‐GS genes in developing grains at DAP20 within the wheat population. C1, C2, C3 are clustered by the Log_2_(TPM value) with the “complete” method using pheatmap. G) Comparison of the SDS sedimentation volume of wheat accessions among three groups. The student's *t*‐test was used to determine the statistical significance between two groups. *, *P* ≤ 0.05; **, *P* ≤ 0.01; ns, no significant difference. H) Scatter plot of gluten content against the expression level values of *TaGlu‐1Ax* in corresponding accessions at DAP20. Each dot denotes one accession, and the line represents the regression trend calculated by the general linear model. I) Comparison of the SNP density in open chromatin regions among each subtype gene. The SNP density indicates the number of SNP located in the ATAC‐seq peak in the Watkins population (SNPs per Kb). Box denote the 25^th^, median, and 75^th^ percentiles, and whiskers indicate the 1.5× interquartile range. The LSD multiple comparisons test was used to determine the significance of CV differences among subtype genes. Different letters indicate a significant difference at *P* ≤ 0.05. J) Scatter plot of the expression CV at DAP10 (brown dots) and DAP20 (cyan dots) against the SNP density of each subtype of genes (SNPs per Kb). Each dot denotes one subtype, and the gray line represents the regression trend calculated by the general linear model. K) Comparison of the −log_10_(*p*‐value) of SNPs with starch content among different genomic features. The LSD multiple comparisons test was used to determine the significant differences among subtype genes. Different letters indicate a significant difference at *P* ≤ 0.05. L) The Pearson correlation coefficient (PCC) of expression level in population between *MNB1A* and SSP and starch synthesis genes. Box denote the 25th, median, and 75th percentiles, and whiskers indicate the 1.5× interquartile range. The LSD multiple comparisons test was used to determine the significance of PCC differences among subtype genes. Different letters indicate a significant difference at *P* ≤ 0.05.

To address whether variability in the expression of Starch and SSP genes contributes to the final starch and storage protein contents, as well as the diversity observed in grain size and quality across wheat varieties, we assessed the coefficient of variation (CV) in the expression of these genes in 102 representative wheat varieties (**Figure** **3A**; Figure S3A, Table S4, Supporting Information). RNA sequencing of developing grains at DAP10 and DAP20 revealed notably higher CV of SSP genes, with *ω*‐gliadins showing the most variability, while HMW‐GS exhibited the least. Among the Starch genes, *TaBt1* and *PHO* exhibited higher expression variations within the wheat population compared with other Starch genes (Figure 3A; Figure S3A, Supporting Information). Interestingly, traits associated with protein content and SSP subunit composition displayed higher CVs than traits related to starch content and pasting properties (Figure S3B, Supporting Information).

We further examined the relationship between the expression levels of different subtypes of Starch and SSP genes, with grain size and quality traits. Starch genes, more active in the early stages of endosperm development, showed a stronger correlation with starch content and pasting properties at DAP10 than at DAP20 (Figure 3B, Table S5, Supporting Information). Conversely, SSP coding genes exhibited a higher correlation with traits related to protein content and SSP subunit composition at DAP20 (Figure 3B). Among the 102 wheat varieties, *TaBt1* expression varied significantly, with the highest in C3, followed by C2, and the lowest in C1 (Figure 3C). Remarkably, the GW of accessions in each cluster followed the order of expression level (C3 > C2 > C1), while setback viscosity exhibited the opposite trend (Figure 3D; Figure S3C, Supporting Information). Correspondingly, *TaBt1‐A1* expression levels were significantly positively correlated with GW and negatively with setback viscosity (Figure 3E). Similarly, the expression level of *TaGlu‐1*, especially *TaGlu‐1Ax*, showed a significant positive correlation with gluten content and sodium dodecyl sulfate (SDS) sedimentation volume in the population (Figure 3F–H; Figure S3D,E, Supporting Information). These findings suggest that the expression variation of SSP and starch synthesis genes may contribute to the observed diversity in grain size and quality within the wheat population.

Considering the crucial role of open chromatin regions in regulating genes related to SSP and starch synthesis, we explored whether natural variations in these regions contribute to expression variations. The SNP density in open chromatin regions of SSP genes, particularly LMW‐GS and *γ*‐gliadins, was higher than that of starch synthesis genes, while AGPS and SS exhibited lower SNP densities (Figure 3I). Consistently, this SNP density in open chromatin regions strongly correlated (*R* > 0.5) with their expression variability among cultivars in the population, both in developing grains at DAP10 and DAP20 (Figure 3J). Additionally, natural variations in open chromatin regions had higher detection power, contributing to phenotypic variation within the population (Figure 3; Figure S3F,G, Supporting Information). A larger proportion of SNPs were significantly associated with starch and protein content in a previously characterized wheat population,^[^ ^56^ ^]^ compared to other genomic regions (Figure 3K; Figure S3F,G, Supporting Information). Collectively, natural variations in the open chromatin regions of SSP and starch synthesis genes may represent causal variations contributing to their expression difference and the observed diversity in grain size and quality within the wheat population.

### 2.4 Unraveling Transcriptional Networks for Storage Production Regulation

**Figure 4:**
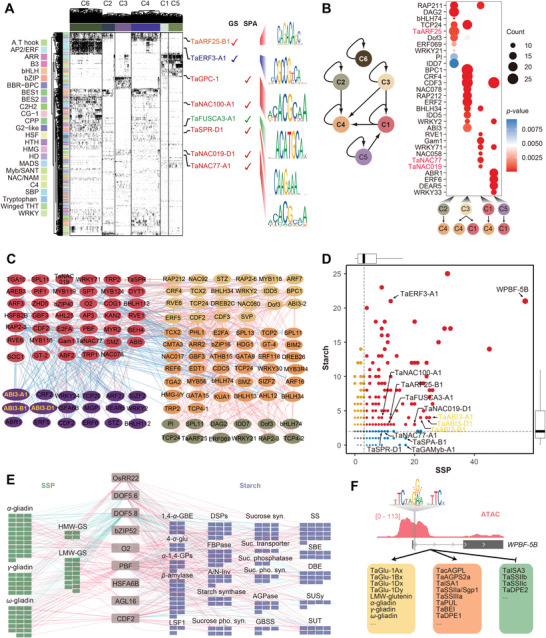
Transcriptional regulatory networks (TRNs) during endosperm development. A) The 2D transcriptional regulatory matrix for TFs‐targets. Each column represented a target gene and each row represented a TF. The target genes were ordered by clustering results in Figure 1D. The black color represents a regulation relationship between TFs and targets, while the white color means no regulation. Known TFs regulating grain size (GS) and storage production accumulation (SPA) were highlighted. The binding motifs of representative TFs were exhibited. B) Transcriptional regulatory relationship between different cluster genes (left) and the enrichment of conducting TFs (right). C) TFs‐TFs regulatory networks among clusters C1, C2, C3, C4, C5. Enriched TFs were shown in color circles. The color code for each cluster was the same as shown in Figure 4B. Known TFs were indicated. D) Different TFs could directly regulate multiple SSP (*x*‐axis) and starch (*y*‐axis) biosynthesis‐related genes. TFs that target over the median of regulated SSP and starch biosynthesis were highlighted in color, with brown, blue, and red dots indicating the TFs enriched for Starch genes, SSP genes, and for both starch and SSP genes, respectively. Known TFs were labeled, and *TaABI3‐A1/B1/D1* were highlighted. The outer boxplot represented the statistics of the number of SSP and starch genes. E) Co‐regulation of starch biosynthesis and SSP composition‐related genes by common TFs. SSP and starch biosynthesis genes were colored in red and blue, respectively. Each function pathway genes were clustered. The red line between TFs and target represented the positive regulation, while the blue line represented the negative regulation. F) Regulation of *WPBF* on starch biosynthesis and SSP composition‐related genes. The proximal accessible region of *WPBF* included an SNP in a TF binding motif. WPBF could positively regulate SSP or starch biosynthesis genes, as shown by the arrow, and negatively regulate starch biosynthesis genes, shown by the break line.

Deciphering the intricate regulatory mechanisms governing starch biosynthesis and SSP accumulation during endosperm development is crucial for comprehending storage production accumulation. To achieve this, we constructed hierarchical transcriptional regulatory networks (TRNs) by integrating gene co‐expression information (GENIE3) and ATAC‐seq footprint data (Figure S4A, Supporting Information). This resulted in a complex TRN with 3187686 connections and 60973 nodes involving 1856 TFs (**Figure** **4A**). TFs of the same family exhibited similar binding motifs and shared common targets, revealing their coordinated regulation (Figure 4A). Notably, known TFs implicated in wheat end‐use quality, such as NAC TFs, TaERF3‐A1, TaFUSCA3‐A1, TaSPR‐D1, TaARF25‐B1, TaNACs, and TaGPC‐1, were identified in the TRNs (Figure 4A).

Furthermore, the TRNs highlighted the conservation of TF binding sites across the different clusters, emphasizing the intricate regulatory relationships governing gene expression during endosperm development (Figure S4B,C, Supporting Information). Genes within cluster1, enriched in protein biosynthesis, exhibited regulation by clusters3 and clusters5, suggesting coordinated control of key processes (Figure 4B,C). Specific enrichment patterns like TaARF25 and NAC TFs for the regulation of cluster4 genes by clusters2 and clusters1, respectively, underscored the nuanced regulatory networks orchestrating endosperm development (Figure 4B). Examining the hierarchical positioning of established TFs associated with storage production, including WPBF, TaSPR, TaSPA, TaGAMyb, TaNAC019, and TaNAC77, provided valuable insights into their direct influence on storage production accumulation (Figure 4C). In addition to several documented TFs known to influence storage production, we identified over a hundred TFs that could exert positive or negative regulation on either or both of the Starch and SSP genes (Figure 4D,E; Figure S4D, Supporting Information). Among those, the majority of TFs show negative regulation for both SSP and starch biosynthesis genes, whereas fewer TFs could simultaneously regulate Starch and SSP genes positively. Furthermore, several TFs could regulate the expression of Starch and SSP genes in opposite ways, such as WPBF, ERFs, MNB1A, and ABI3, suggesting their potential function in balancing SSP and starch biosynthesis (Figure S4D, Supporting Information).

Notably, WPBF emerged as a central player in this regulatory network, displaying a remarkable capacity to simultaneously positively and negatively regulate a multitude of storage production genes (Figure 4D,E). For instance, WPBF may positively regulate SSP such as HMW‐GS, LMW‐GS, and gliadins genes, positively regulate certain starch biosynthesis genes, such as *TaAGPase*, *TaISA*, *TaBEI*, while negatively regulate others, such as *TaISA3, TaSSII, TaDPE2*. The identification of an SNP at the binding site of WPBF within the wheat population added an additional layer of significance, suggesting potential variations in its direct regulatory impact (Figure 4F).

Therefore, the generated TRN enhances understanding of transcriptional regulation of starch biosynthesis and SSP accumulation. Relationships among TFs, binding motifs, and genetic variations within the population offer insights for optimizing storage production.

### 2.5 Identification of Novel Regulators for Grain Yield and Quality in Wheat

**Figure 5:**
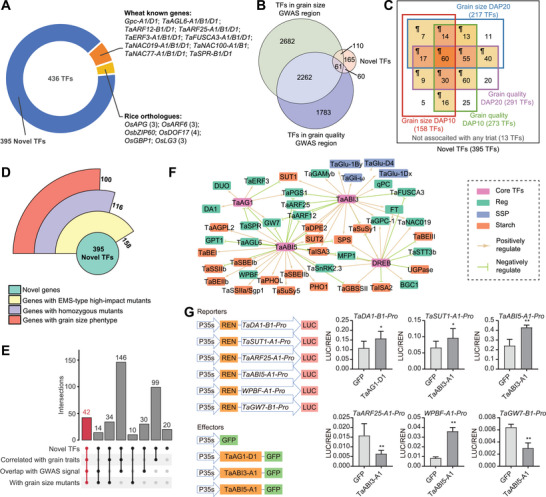
Filter and validation of potential key TFs in shaping grain development traits. A) Characteristic of core TFs from TRN analysis. B) Venn diagram showing the overlapping of novel TFs identified from TRN with TFs located in the interval of significant GWAS signal for grain size and grain quality. C) Summary of novel TFs identified from TRN showing an association between TFs’ expression with grain size and quality traits variation in a core collection of wheat accessions. The genes identified in more than two groups were marked with background color, with genes overlapped in more groups in darker color, the genes reserved for further analysis. D) Summary of novel TFs with different categories of KN9204 TILLING mutant lines. E) Overlapping of the novel TFs supported by GWAS signal, expression‐phenotype correlation, and morphological defects of TILLING mutants. F) A case showing of TRN module containing core TFs such as TaABI3, TaABI5, TaDREB, TaAG, and the potential targets involved in SSP and starch biosynthesis, as well as the known regulators. Color codes are as indicated. G) Dual luciferase reporter assays showing certain transcriptional regulatory circuits between TFs and their targets in the predicted TRN. Student's *t*‐test was used for the statistical significance. *, *P* ≤ 0.05; **, *P* ≤ 0.01.

Based on the constructed TRN, a substantial portion of known TFs involved in the structural organization and direct regulation of storage production biosynthesis genes was identified (Figure 4C,D). The integration of these two gene lists yielded a total of 436 core TFs (**Figure** **5A**, Table S6, Supporting Information). Among them, 26 TFs have been functionally validated in wheat for their role in regulating grain size or seed quality, including factors like *TaGPC‐1*,^[^ ^57^ ^]^ *TaNAC019*,^[^ ^11^, ^12^ ^]^ and *TaSPR*.^[^ ^16^ ^]^ Additionally, 15 TFs are orthologous to regulators for kernel development in rice, such as *OsbZIP60*,^[^ ^58^, ^59^ ^]^ *OsDOF17* ^[^ ^60^ ^]^ and *OsAPG*.^[^ ^61^ ^]^ Notably, the majority of the TFs, comprising 395 candidates, are novel and have unknown functions in wheat, with enrichment in ERF, NAC, ARF, and B3 TF families (Table S6, Supporting Information).

To assess the efficacy of identifying novel regulators through TRN, we conducted a comprehensive evaluation in three aspects. First, we combined available GWAS data for grain size and quality (Table S7, Supporting Information) with the 395 novel candidates, resulting 231 TFs within 3 Mb regions centered around GWAS signals, with 171 and 121 TFs for grain size and grain quality‐related traits, respectively (Figure 5B, Table S8, Supporting Information). Second, we correlated the expression levels of 395 novel candidate genes at DAP10 and DAP20 with grain size and quality‐related traits in the 102 representative wheat varieties. For 158 TFs, the expression level at DAP10 was significantly correlated with at least one grain size‐related trait (GS‐DAP10 group). Similarly, 273, 217, and 291 TFs were found in the GQ‐DAP10 group, GS‐DAP20 group and GQ‐DAP20 group, respectively (Figure 5C, Tables S8 and S9, Supporting Information). In total, we identified 321 TFs showing a significant correlation in more than two groups (Figure 5C, Table S9, Supporting Information). Third, we conducted a robust investigation of grain size changes in gene‐indexed EMS mutant lines in hexaploid wheat *cv*. KN9204.^[^ ^62^ ^]^ Of the 395 novel TFs, 158 TFs were found to have at least one mutant line that containing loss‐of‐function mutation, and homozygous mutant lines of 100 out of 116 TFs (86.21%) exhibited altered grain size or morphology (Figure 5D, Table S10, Supporting Information). Collectively, a substantial portion of the identified novel TFs exhibited associations with grain‐related traits across genome variation, transcriptional divergence, and protein function levels.

By integrating these verification strategies, 42 novel TFs supported by all approaches were identified for in‐depth study (Figure 5E, Table S11, Supporting Information). Focusing on the TRN for these 42 key TFs, a transcription regulatory module involving TaABI3, TaAG1, TaDREB and TaABI5 was revealed, synergistically regulating grain size regulators (e.g., *TaGW7* and *TaARF12*), starch biosynthesis genes (e.g., *TaSUT1*, *TaDPE2*, and *TaSPS*), and storage protein formation genes (e.g., *TaGlu‐1By*, *TaGlu‐D4* and *ω*‐gliadin genes) (Figure 5F). We further validated certain transcriptional regulatory modules through luciferase reporter assay in tobacco leaves, such as the activation of *TaDA1‐B1* by TaAG1‐D1, activation of *TaSUT1‐A1* and *TaABI5‐A1* while repression of *TaARF25‐A1* by TaABI3‐A1, as well as the activation of *WPBF‐A1* while repression of *TaGW7‐B1* by TaABI5‐A1 (Figure 5G). These findings underscore the accuracy and reliability of predicted grain development regulators by the TRN.

### 2.6 TaABI3‐A1 Negatively Modulates Starch Biosynthesis while Activating Storage Proteins Accumulation

**Figure 6:**
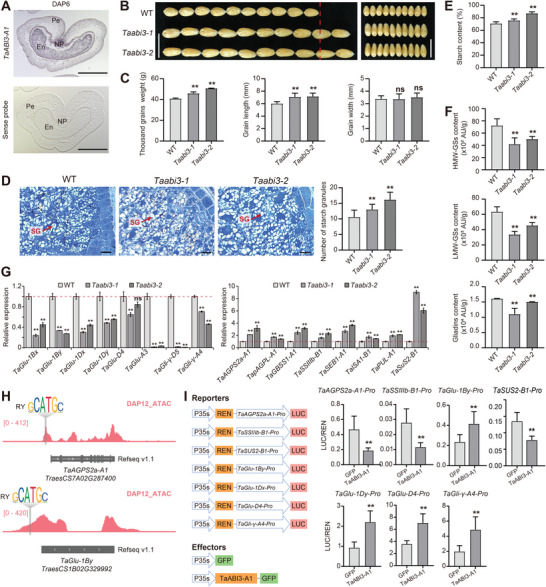
TaABI3‐A1 regulates the grain size and quality of wheat. A) Spatiotemporal expression pattern of *TaABI3‐A1* in DAP6 grain as indicated by in situ hybridization. Sense probe served as a negative control. En, endosperm, Pe, pericarp, NP, nucellar projection. Scale bars = 100 µm. B) Loss‐of‐function *Taabi3‐a/b/d* mutants significantly increases the grain size in wheat. The red line indicates the length of ten wild‐type seeds. C) Quantification of grain size‐related traits between the wild‐type plants and *Taabi3‐a/b/d* mutant lines. Student's *t*‐test was used to determine the difference significance between *Taabi3‐a/b/d* mutant and wild‐type. *, *P* ≤ 0.05; **, *P* ≤ 0.01; ns, no significant difference. D) Representative cross sections of DAP30 grains for wild‐type and *Taabi3‐a/b/d* mutant lines (left panel). Red arrows indicate the starch granules (SG). The quantification of SG numbers is shown in the bar graph (right panel). Student's *t*‐test was used to determine the difference significance between *Taabi3‐a/b/d* mutant and wild‐type. *, *P* ≤ 0.05; **, *P* ≤ 0.01. E,F) Comparison of the content of starch (E) and SSP components (F) between wild‐type and *Taabi3‐a/b/d* mutant lines. Three repeats were carried out for each sample to measure the amounts of starch, HMW‐GSs, LMW‐GSs, and gliadins content. G) The relative expression level of SSRGs and SSP‐related genes in wild‐type and *Taabi3* mutant lines. qRT‐PCR data were normalized to *TaActin*, with values from three replicates shown as mean ± SD. Statistical significance was determined by Student's *t*‐test. *, *P* ≤ 0.05; **, *P* ≤ 0.01; ns, no significant difference. H) IGV screenshot showing the accessible chromatin regions of *TaAGPS2a‐A1*, and *TaGlu‐1By* with a gray vertical line indicating the RY motif. I) Dual luciferase reporter assays showing the transcriptional regulation of TaABI3‐A1 on SSRGs and SSP‐related genes. Schematic diagram showing the vectors used. The relative value of LUC/REN was indicated. Relative LUC/REN values from six replicates shown as mean ± SD. Statistical significance was determined by Student's *t*‐test. *, *P* ≤ 0.05; **, *P* ≤ 0.01.

To delve into the functional insights of key potential factors identified within the TRN in wheat endosperm development, we conducted an in‐depth investigation of a novel regulator, TaABI3‐A1 (*TraesCS3A02G417300*). Through in situ hybridization, *TaABI3‐A1* displayed distinctive expression patterns during grain development, with a higher expression level, particularly in the endosperm and aleurone layer, but lower expression levels in the seed coat at DAP6 (**Figure** **6A**). Three homologous genes of *TaABI3*, with highly similar protein sequences, displayed comparable expression patterns throughout wheat endosperm development stages (Figure S5A,B, Supporting Information). Thus, to elucidate TaABI3's role in wheat grain development, we simultaneously editing the three homoeoalleles using CRISPR/Cas9 in wheat *cv*. JM5265, with a sgRNA targeting conserved domain between homologous genes (Figure S5C, Supporting Information). We obtained two independent *TaABI3‐a/b/d* mutant lines and weak mutant lines *Taabi3‐a/B/D* and *Taabi3‐A/b/d*.

Field growth evaluations of grain size phenotypes for *Taabi3‐a/b/d* revealed a significant increase in GL and TGW in *Taabi3‐a/b/d* compared to the wild‐type JM5265, without changes in grain width (Figure 6B,C). To investigate the underlying mechanisms, we examined endosperm cells in developing seeds at DAP15. Coomassie brilliant blue staining revealed fewer starch granules in *Taabi3‐a/b/d* endosperm cells compared to the wild‐type (Figure 6D), alongside increased total starch content in mature seeds (Figure 6E). Additionally, reverse‐phase high‐performance liquid chromatography (RP‐HPLC) indicated reduced levels of HMW‐GS, LMW‐GS, and gliadins in *Taabi3‐a/b/d* mutants (Figure 6F), highlighting TaABI3's role in regulating SSP. Neither *Taabi3‐a/B/D* nor *Taabi3‐A/b/d* mutant showed significant effects on grain size (TGW, grain length, and grain width), SSP synthesis/accumulation (HMW‐GS content, LMW‐GS content, and gliadins content), or starch content (Figure S5D, Supporting Information). This suggests that homologous of TaABI3 may be functionally redundant, and single mutant or double mutant are insufficient to alter grain size or cause endosperm developmental defects.

Consistence with the grain developmental defects, the *Taabi3‐a/b/d* mutants showed significantly reduced expression levels of genes related to SSP, with *TaGli‐γ‐D5* and *TaGlu‐A3* experiencing drastic declines by dozens of times (Figure 6G). In contrast, key genes controlling sugar or starch synthesis were up‐regulated in the *Taabi3‐a/b/d* mutants compared to the wild‐type (Figure 6G). Notably, the *Wheat sucrose synthase 2* (*TaSUS2‐B1*), significantly associated with TGW in wheat,^[^ ^63^ ^]^ exhibited over six times higher expression in *Taabi3‐a/b/d* mutants than in the wild‐type (Figure 6G). To explore the regulatory mechanism, we utilized ATAC‐seq data and identified the RY motif recognition by B3 family TF, including TaABI3, in the promoters of these starch and SSP biosynthesis‐related genes, such as *TaAGPS2a‐A1* and *TaGlu‐1By* (Figure 6H). Furthermore, a dual luciferase transcriptional activity assay revealed positive transcriptional regulatory of TaABI3‐A1 on SSP‐related genes *TaGlu‐1By*, *TaGlu‐1Dx*, *TaGlu‐D4*, and *TaGli‐γ‐A4*, while it suppressed the expression of *TaAGPS2a‐A1* and *TaSSIIIb‐B1*, in tobacco leaves (Figure 6I; Figure S5E, Supporting Information).

In summary, TaABI3‐A1 negatively regulates the grain size primarily by inhibiting starch biosynthesis transcriptionally, while promoting storage protein accumulation through the activation of SSP‐related genes.

### 2.7 Genetic Variation of *TaABI3‐A1* Associates with Grain Weight and Quality

**Figure 7:**
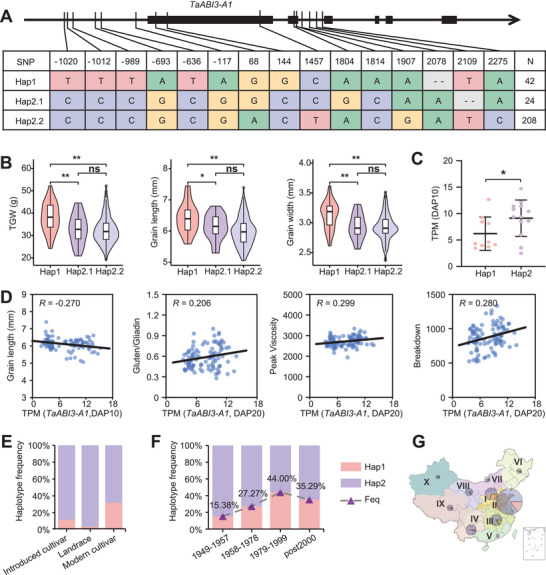
Haplotype analysis of *TaABI3‐A1* and breeding selection of elite allele. A) Schematic diagram showing the polymorphism for each haplotype of *TaABI3‐A1* in the Chinese wheat mini‐core collection (MCC) wheat collection. The coordinate is related to the transcription start site. B) Violin plot indicating the comparison of grain size‐related traits among wheat accession with different haplotypes of *TaABI3‐A1*. The student's *t*‐test was used to determine the statistical significance between the two groups. *, *P* ≤ 0.05; **, *P *≤ 0.01; ns, no significant difference. C) Comparison of the *TaABI3‐A1* expression level between Hap1 and Hap2. In the 102 representative wheat varieties for population transcriptome, 23 accessions were with public available whole genome re‐sequencing data and used for analysis. D) Scatter plot showing the correlation between grain length, peak viscosity, breakdown value, and gluten: gliadin ratio with the expression of *TaABI3‐A1*. Each dot denotes one accession, and the black line represents the regression trend calculated by the general linear model. E,F) The percentages of accessions carrying different haplotype of *TaABI3‐A1* in categories (E) and during the different breeding processes in China (F). G) The percentage of accessions carrying different haplotypes of *TaABI3‐A1* in each ecological zones of China. The size of pie charts in the geographical map shows the number of accessions, with percentages of the two alleles in different colors (Hap1, pink; Hap2, purple).

To explore the link between genetic variation in *TaABI3‐A1* and grain‐related traits in wheat, we examined allelic diversity in the Chinese wheat mini‐core collection (MCC), a panel of 287 representative varieties.^[^ ^36^ ^]^ In the *TaABI3‐A1* genomic region, we identified 15 polymorphic sites, including six SNPs in the promoter, five SNPs on the exon, and two SNPs with two 1‐bp deletions in the intron, forming three haplotypes (**Figure** **7A**). Hap1, represented by 42 accessions, exhibited significantly greater grain length, width, and weight compared to Hap2.1 and Hap2.2, which showed no discernible differences, designating it as the excellent haplotype (Figure 7B, Table S12, Supporting Information).

Interestingly, as indicated in the population transcriptome (Figure 3), Hap1 displayed lower expression levels compared to Hap2 (Figure 7C), while Hap2.1 and Hap2.2 grouped together due to the similarity in grain morphology traits. Moreover, we observed a notable negative correlation of TaABI3‐A1 expression with grain length, alongside a positive correlation with flour quality traits such as peak viscosity, breakdown value, and gluten: gliadin ratio (Figure 7D). These findings imply that distinct haplotypes of *TaABI3‐A1* correlate with its expression, thereby influencing variations in grain size and quality among wheat varieties, affecting various traits to varying degrees.

To assess whether the excellent Hap1 was selected in China's breeding process, we analyzed haplotype frequency in the MCC population. The percentage of accessions with the Hap1 was notably higher in modern cultivars than in landraces and introduced cultivars from other countries (Figure 7E). Interestingly, the frequency of Hap1 steadily increased from 15.38% in 1949–1957 to 44.00% in 1978–1999, slightly decreasing in post‐2000 cultivars (Figure 7F). Additionally, distinct haplotype distributions were observed in major Chinese agro‐ecological zones, with zones I, II, and III showing a higher Hap1 frequency than other zones (Figure 7G). Collectively, the bigger grain Hap1 of *TaABI3‐A1* was selected during the breeding process in China.

## 3 Discussion

Understanding the molecular mechanisms governing starch biosynthesis and SSP accumulation is vital for breeding improved wheat varieties.^[^ ^5^, ^64^ ^]^ Efforts to identify enzymes for starch and SSP, along with genetic variations associated with them, have linked to enhanced grain quality.^[^ ^29^, ^31^ ^]^ However, a gap exists due to the lack of systematic transcriptional regulation analysis of starch and SSP coding genes. In this study, we generated transcriptome and epigenetic profiles across key endosperm development stages (Figure 1). Our approach identified specific open chromatin regions regulating starch and SSP genes (Figure 2). By integrating expression data and *cis*–*trans* regulation, we built a hierarchical transcriptional regulatory network (TRN) for starch and SSP (Figure 4), efficiently identifying novel regulators for grain yield and quality (Figure 5).

### 3.1 Significance of Epigenetic Regulatory Regions in Shaping Starch and SSP Diversity

Various steps are essential for producing starch and different gluten proteins with distinct characteristics, and previous efforts have identified enzymes and non‐enzymatic factors involved in this process.^[^ ^65^, ^66^, ^67^ ^]^ Studies have explored coding variations in these genes contributing to diverse starch and SSP products.^[^ ^68^, ^69^ ^]^ Our research reveals that dynamic epigenetic features, particularly chromatin accessibility, H3K27ac, and H3K27me3, collectively regulate SSP genes, with a less pronounced effect of H3K27me3 on starch biosynthesis genes (Figure 2). In addition to coding region variations, open chromatin regions in the promoter play a crucial role as a genetic variation resource influencing diversity in grain yield and quality traits among wheat accessions (Figure 3). These open chromatin regions significantly impact the transcriptional regulation circuit (Figure 2), particularly by altering TFs binding motifs within these regions. DNA variations in *cis‐*motifs likely change the recognition of specific TFs, influencing transcriptional regulation activity (Figure 3). Indeed, genetic variation within ATAC‐seq peaks leads to varied expression of starch and SSP coding genes, ultimately shaping grain yield and quality traits (Figure 3).

### 3.2 Comprehensive TRN Facilitates Systematic Identification of Regulators Governing Starch and SSP Biosynthesis

Utilizing time‐serial RNA‐seq and chromatin accessibility data from developing endosperm tissue, we constructed a TRN encompassing starch biosynthesis and SSP accumulation processes (Figure 4). Among the 436 core TFs within the TRN, we identified numerous functionally characterized factors involved in grain weight or quality, affirming the TRN's ability to pinpoint crucial regulators for starch and SSP biosynthesis. Notably, a significant portion of the 395 potential novel regulators met individual criteria for functional validation through loss‐of‐function mutants, expression‐phenotype association analysis in a core collection of wheat accessions, or overlapping analysis with GWAS signals associated with grain size and quality traits. Moreover, 42 TFs appeared in all three screening methods. The collective data reinforces the efficiency and accuracy of our integration strategy in the systematic identification of novel regulators governing starch and SSP biosynthesis in wheat.

### 3.3 TaABI3‐A1, a Promising Candidate for Balanced Grain Yield and Enhanced Quality in Wheat Breeding

In our investigation of 42 high‐confidence novel regulators, TaABI3‐A1 stood out and underwent detailed functional analysis through the generation of knock‐out mutants via genome editing. TaABI3‐A1 was found to positively activate SSP coding genes, including *TaGlu‐1*, *TaGlu‐D4*, and *TaGli‐γ‐D5*, resulting in lower accumulation of HMW‐GS, LMW‐GS, and gliadins in *Taabi3* mutants. Conversely, it inhibits various starch biosynthesis enzyme coding genes, leading to increased TGW and GL when in a loss‐of‐function state. Notably, in a collection of ≈300 wheat accessions, an elite haplotype of *TaABI3‐A1* was identified, exhibiting higher TGW and grain size. Importantly, this elite haplotype has been selected during the breeding process in China likely with the pursuit of increasing grain yield, especially from 1949–1999 and particularly in zones I, II, and III, where with a higher breeding selection intensity and variety renewal rates.^[^ ^70^ ^]^ The superior grain yield is associated with the alteration of *TaABI3‐A1* expression level. Higher TaABI3 expression is linked to lower GL but improved flour quality, as evidenced by increased peak viscosity, breakdown value, and gluten: gliadin ratio. This suggests the possibility of precisely manipulating the temporal expression level of *TaABI3‐A1* to achieve a balance between grain yield and seed quality, especially given the slight temporal differences in starch biosynthesis and SSP accumulation during the grain‐filling window.

### 3.4 Valuable Data Source for Mining Regulators in Shaping Grain Development

In addition to the *TaABI3‐A1* case, we have identified 41 other high‐confidence novel candidates with the potential to regulate starch and SSP biosynthesis in wheat. Further research is warranted‐thoroughly characterize the functions of these factors. Additionally, exploring natural variations at these gene loci could serve as a valuable resource for breeding improved wheat varieties with superior grain weight and quality, especially when aiming to uncouple the regulation of starch biosynthesis and SSP accumulation. From a broader perspective, the time‐course transcriptome and epigenetic dataset as well as the population transcriptome of developing endosperm generated in this study can benefit the research community by facilitating the swift verification of potential candidates. It also provides guidance for studying transcriptional regulatory networks and offers information on development‐dynamic open chromatin regions for fine‐tuning gene expression, proving useful for precision genome editing in breeding applications.

## 4 Experimental Section

### Plant Materials and Growth Conditions

The wheat cultivar Chinese Spring was used in this study. The seedlings were planted in soil and grown in the greenhouse at 22 °C/20 °C day/night, under long‐day conditions (16 h light/8 h dark). The stamens were removed before the pollen maturation. Then, artificial pollination was conducted and recorded the number of days to ensure the accurate time of seed development. The seeds at DAP0, DAP2, DAP4, DAP6, DAP8, DAP12, DAP16, and DAP22 stages were sampled for later total RNA extraction and nuclei isolation. The dissection method of endosperm following the instructions in the previous report with some modifications.^[^ ^45^ ^]^ Embryo sacs in DAP0, DAP2, and DAP4 were dissected in a 5% Sucrose solution containing 0.1% RNase inhibitor with fine forceps using the dissecting microscope. For DAP6 and later stages, the relatively independent embryo and seed coat were detached using the same method and dropped, with endosperm left and collected. Endosperm and embryo sacs sampled from about eight spikes were pooled for one biological replicate in early stages and three to five were pooled for one biological replicate in late stages. The RNA‐seq (three replicates), ATAC‐seq, and CUT&Tag (two replicates) experiments at eight or six development stages were carried out.

### Generation and Genotyping of *TaABI3 CRISPR/Cas9* Lines

To knock out *TaABI3* in wheat *cv*. JM5265, the sgRNA 5′‐TCGCCAACTGGATCCTACGG‐3′ located in the first exon was used. The CRISPR/Cas9 editing was conducted in Genovo Biotechnology Co. (Beijing, China) following the methods as previously reported.^[^ ^71^ ^]^ To identify mutations in *TaABI3‐A1* (*TraesCS3A02G417300*), *TaABI3‐B1* (*TraesCS3B02G452200*), and *TaABI3‐D1* (*TraesCS3D02G412800*), gene‐specific primers were designed around the sgRNA target site. PCR products were genotyped by Sanger sequencing. Primers used for genotyping are listed in Table S13 (Supporting Information).

### Grain Development Traits Measurement

A Wanshen SC‐G seed detector (Hangzhou Wanshen Detection Technology Co., Ltd.) was used to measure grain width, grain length, and TGW, with grains from each plant randomly sampled for three replicates. For starch content measurement, 100 mg flour was used and the content was measured using the Megazyme Total Starch Assay Kit (Megazyme; KTSTA‐50A) following the manufacturer's method (three replicates per sample). For the SSP content determination, the contents of HMW‐GSs, LMW‐GSs, and gliadins were detected by RP‐HPLC as previously described (three replicates per sample).^[^ ^12^ ^]^ The Agilent Technologies 1260 Infinity IIRP‐HPLC system and an Agilent ZORBAX 300SB‐C18 column (150 mm × 4.6 mm, 5 µm) were used. Water containing 0.6 mL L^−1^ trifluoroacetic acid (solvent A) and acetonitrile containing 0.6 mL L^−1^ trifluoroacetic acid (solvent B) were used as elution solvents. The column temperature was 60 °C and the flow rate was 1 mL min^−1^. The injection volume was 8 µL and the eluent was monitored at 210 nm. The total amounts of HMW‐GSs, LMW‐GSs, and gliadins were estimated by integrating the relevant RP‐HPLC peaks present in the chromatograms.

### Semi‐Thin Sections and Coomassie Brilliant Blue (CBB) Staining

Wild‐type and *Taabi3* mutant seeds at DAP15 were fixed in FAA solution (63% ethanol, 5% acetic acid, 5% formaldehyde) under vacuum for 2 h and incubated for 24 h at room temperature. The samples were dehydrated through a graded ethanol series and embedded in Technovit 7100 resin (Kulzer), according to the manufacturer's instructions. Sections (2‐µm thick) were cut using a UC7&2265 microtome (Leica).To visualize starch granules and protein bodies, the sections were stained with 0.1% w/v coomassie brilliant blue R‐250.^[^ ^72^ ^]^ Images of stained sections were captured using an OLYMPUS DP74 Microscope. The endosperm starch granules number was manually calculated for six seeds.

### In Situ Hybridization Assay

RNA in situ hybridization was carried out as described previously.^[^ ^45^ ^]^ Fresh seeds were fixed in formalin‐acetic acid‐alcohol overnight at 4 °C, dehydrated through a standard ethanol series, embedded in Paraplast Plus tissue‐embedding medium (Sigma‐Aldrich, P3683), and sectioned at 8 µm width using a microtome (Leica Microsystems, RM2235). Digoxigenin‐labeled sense and antisense RNA probes based on the sequence of *TaABI3‐A1* were synthesized using a DIG Northern Starter Kit (Roche, 11 277 073 910), according to the manufacturer's instructions. Primers used for the sense and antisense probe synthesis are listed in Table S13 (Supporting Information).

### Luciferase Reporter Assays

To generate pTaDA1‐B1::LUC, pTaSUT1‐A1::LUC, pTaABI5‐A1::LUC, pTaARF25‐A1::LUC, pWPBF‐A1::LUC, pTaGW7‐B1::LUC, pTaAGPS2a‐A1::LUC, pTaSSIIIb‐B1::LUC, pTaGlu‐1By::LUC, pTaGlu‐1Dy::LUC, pTaGlu‐D4::LUC, pTaGli‐*γ*‐A4::LUC and pTaSUS2‐B1::LUC, 2‐Kb promoter fragments upstream of each gene from cv were amplified. Chinese Spring and ligated them with the CP461‐LUC as the reporter vector. The ORFs of TaAG1‐D1, TaABI3‐A1, and TaABI5‐A1 were cloned into the Psuper‐GFP vector as effectors, and these plasmids were transformed into GV3101 and injected into *N. benthamiana* leaves in different combinations. Dual luciferase assay reagents (Promega, VPE1910) with the Renilla luciferase gene as an internal control were used for luciferase imaging. The Dual‐Luciferase Reporter Assay System kit (Cat#E2940, Promega) was used to quantify fluorescence signals. Relative LUC activity was calculated by the ratio of LUC/REN. The relevant primers are listed in Table S13 (Supporting Information).

### RNA Extraction, qRT‐PCR Analysis, RNA Sequencing

Total RNA was extracted using HiPure Plant RNA Mini Kit (Magen, R4111‐02). First‐strand cDNA was synthesized from 2 µg of DNase I‐treated total RNA using the TransScript First Strand cDNA Synthesis SuperMix Kit (TransGen, AT301‐02). qRT‐PCR was performed using the ChamQ Universal SYBR qPCR Master Mix (Vazyme, Q711‐02) by QuantStudio5 (Applied biosystems). The expression of interested genes was normalized to *TaActin* for calibration, and the relative expression level was calculated via the 2^−ΔΔCT^ analysis method.^[^ ^72^ ^]^ Primers used for qRT‐PCR are listed in Table S13 (Supporting Information).

Paired‐end RNA‐seq libraries were prepared and sequenced via the Illumina NovaSeq platform according to the manufacturer's standard protocols by Annoroad Gene Technology.

### CUT&Tag and ATAC‐Seq Experiment

Embryo sacs and endosperm of DAP0, DAP2, DAP4, DAP6, DAP8, DAP12, DAP16, and DAP22 stages were used to isolate nuclei and carried out the CUT&Tag and/or ATAC‐seq experiments. CUT&Tag and ATAC‐seq experiments followed the previously described method.^[^ ^45^ ^]^ The fresh samples were used to isolate nuclei, and the appropriate number of nuclei (1000–50 000) were resuspended by the corresponding antibody and secondary antibody. Then the nuclei were incubated with pA‐Tn5. *Tn5* transposase used and a tagmentation assay was done following the manual (Vazyme, TD501‐01). Libraries were purified with AMPure beads (Beckman, A63881) and sequenced using the Illumina Novaseq platform at Annoroad Gene Technology. Antibodies used for histone modifications are listed in Table S13 (Supporting Information).

### Data Quality Control and Alignment

For the analysis of RNA‐seq, CUT&Tag, and ATAC‐seq data, data quality control was first conducted using faster software.^[^ ^73^ ^]^ This step ensured the removal of low‐quality reads and adapter sequences, resulting in clean datasets for further processing. The cleaned datasets were then aligned to the Chinese Spring reference genome IWGSC RefSeq v1.1 assembly.^[^ ^74^ ^]^ For RNA‐seq data, the alignment was performed using hisat2,^[^ ^75^ ^]^ while for CUT&Tag and ATAC‐seq data, bwa was employed.^[^ ^76^ ^]^

### RNA‐Seq Data Processing

The raw count of reads of each gene was calculated using FeatureCount software (Liao et al., 2014). The raw counts were normalized to TPM. The genes with TPM ≥ 0.5 were defined as expressed genes. The relative expression of each homologous gene in the triad was then normalized to the TPM expression of that gene divided by the total TPM expression of the triad. The identification of balance and bias expression of trads followed the previously published strategy.^[^ ^74^ ^]^

DEGs in RNA‐seq data were identified using edgeR to compare different samples.^[^ ^77^ ^]^ A threshold absolute value of Log_2_(Fold Change) ≥ 1 and FDR ≤ 0.05 was used for DEGs calling. k‐means clustering algorithm was employed for gene clustering, enabling to categorize genes into meaningful groups based on their expression patterns. Function enrichment analysis were done using an R package ClusterProfiler.^[^ ^78^ ^]^

### Cut&Tag and ATAC‑Seq Data Processing

Peak calling for CUT&Tag and ATAC‐seq data was conducted using macs2.^[^ ^79^ ^]^ Given the diverse nature of histone modifications, different parameters were applied for peak calling based on the type of modification. For histone modifications such as H3K27ac and H3K4me3, which were characterized as narrow peaks, we used the parameters “‐p 1e‐3 ‐f BAMPE –keep‐dup all” was used. In the case of broad peak histone modifications like H3K27me3, the parameters “—broad –broad‐cutoff 0.05 ‐f BAMPE –keep‐dup all”. For ATAC‐seq, the peak calling was performed with the parameters “‐f BAMPE –keep‐dup all –cutoff‐analysis –nomodel –shift −100 –extsize 200”. Peaks were assigned to the nearest genes using Chipseeker.^[^ ^80^ ^]^ The reads under peaks were calculated using FeatureCount,^[^ ^81^ ^]^ and the raw counts were normalized to CPM value.

For ATAC‐seq data, the TFs binding activity was calculated by R package chromVAR (v1.10.0).^[^ ^82^ ^]^ HINT (Hmm‐based IdeNtification of TF footprints) was used for ATAC‐seq footprints identity.^[^ ^83^ ^]^ JASPAR Plantae database (https://jaspar.genereg.net/) was used as a motif set.^[^ ^84^ ^]^ Custom wheat genomes were configurated based on the introduction of HINT software using the Chinese Spring reference genome IWGSC RefSeq v1.1.

### Transcriptional Regulatory Network (TRN) Construction and Key TFs Identification

To construct a robust transcriptional regulatory network, RNA‐seq and ATAC‐seq data were integrated^[^ ^45^, ^85^, ^86^, ^87^ ^]^ This comprehensive approach allowed the authors to map TF interactions and gene regulatory relationships in wheat.

Diamond was first used to perform blast searches, aligning plant TF protein sequences from the JASPAR database with wheat protein sequences which identified TFs‐Motif relationships in wheat. For each gene, accessible chromatin regions (pACRs) sequences were analyzed using ATAC‐seq footprint analysis which generated the Motif‐targets relationships. Combining the TFs‐Motif and Motif‐targets data, we derived the TFs‐targets relationships were derived. Using the GENIE3 algorithm, transcriptional correlations between wheat TFs and all genes based on RNA‐seq data were analyzed. Finally, for each TF, the transcriptional correlation of genes were compared with and without the TF footprint in their promoter regions. This comparison was used to calculate the significance of TFs‐target relationships.

Core TFs were identified in wheat endosperm development, focusing on two categories: structure TFs and TFs directly upstream of storage production genes. The TFs significantly enriched in the TFs‐TFs regulatory network were considered as the structure TFs. The upstream regulators of protein and starch synthesis genes were considered as directly upstream of storage production genes.

### Haplotype Analysis of *TaABI3‐A1*

Natural variation retrieved from the whole‐exome sequencing project of the Chinese wheat mini‐core collection,^[^ ^36^ ^]^ composed of 287 representative selected varieties for the Chinese national collection, were used to assess the allelic variation of *TaABI3‐A1*. The polymorphism with missing rate < 0.5, min allele frequency > 0.05, and heterozygosity < 0.5 were retained for further haplotype analysis using Haploview 4.2, and the differences of the grain size phenotypes corresponding to different haplotypes were tested. The haplotype frequency in each breeding process of China and among the major Chinese agro‐ecological zones was calculated according to the material information provided.^[^ ^36^ ^]^

### Field Growth Condition and Grain Development Traits Analysis of 102 Wheat Accessions

A total of 102 accessions with abundant phenotypic variation in grain size and quality traits, from the Yellow and Huai Valley winter wheat region and representing the genetic structure of a previously characterized common wheat population, were selected as a core wheat collection.^[^ ^88^ ^]^ The collection was sown with two replicates, with five 150‐cm‐long rows for each accession, in a field at Beijing (39°54′N, 116°25′E) during crop season 2017–2018. Field experiments were performed using a completely randomized design, and agronomic management followed local practices.

Mature grains of each accession were harvested and air‐dried to water content between 11–13%. After moistening for 10 h, grains were tempered and milled with a Brabender Junior mill (MLU 220, Uzvil, Switzerland) using method AACC 26‐21A, and the reserved flour after screened with a 70GG sieve was used to conduct further phenotypic assay of the content of starch, gluten, gliadin, and total storage protein, SDS sedimentation volume, pasting characteristics of starch and dough quality parameters.

### Population RNA‐Seq and Transcriptome Analysis of 102 Wheat Accessions

The flowering spikes of each accession were marked with a sign, and six spikes were harvested at DAP10 and DAP20, respectively. The grains in the middle of the spikes were sampled, immediately frozen in liquid nitrogen, and then stored at −80 °C. The grains of each accession at DAP10 or DAP20 were mixed and divided into two independent biological replicates. Total RNA was extracted using a TRIzol kit (Invitrogen, Carlsbad, CA, USA), and high‐quality RNA samples detected by the Agilent2100 Bioanalyzer instrument were subjected‐PE150 sequencing using a BGISEQ500 platform (BGI, Shenzhen, China). Filtered reads were mapped to the wheat reference genome IWGSC Refseq v1.1,^[^ ^70^ ^]^ and transcripts aligned to each gene were calculated and normalized to TPM values.

### Coefficient of Variation and Correlation Analysis

The SNP density and CV were used as a measure of genotypic variability, transcriptional variability, and phenotypic variability. Genotypic variability was characterized by SNP density. It was calculated using the SNP number divided by the length genomic region, with a percentage of 1% indicates that there was a variation within the population every 100 bp. The CV was used to assess the variability of expression level and phenotypic traits, defined as the standard deviation divided by the mean. CV, a unitless statistical measurement independent of size, was widely used to measure and compare variation of quantitative traits, evolvability, and phenotypic plasticity.^[^ ^89^ ^]^

To calculate the correlation between gene expression levels and epigenetic modification signals, Pearson correlation coefficients were calculated between the *z*‐scale TPM values and the *z*‐scale CPM values of promoter region (from −3000 bp to +1000 bp) annotation peaks across each gene. To calculate the correlation between gene expression levels and phenotypic traits, Pearson correlation coefficients were calculated between the gene expression TPM values and trait value of wheat varieties. For the expression correlation between two genes, TPM values in each wheat variety were used to calculate the Pearson correlation coefficients.

### Statistics and Data Visualization

If not specified, R (https://cran.r-project.org/; version Kulzer4.0.2) was used to compute statistics and generate plots. For the two groups’ comparison of data, the student's *t*‐test was used, such as Figures 3, 5, 6, 7 and Figures S3C,D and S5D. For three or more independent groups comparison of data, Fisher's Least Significant Difference was used, such as Figures 2, 3 and Figures S3A,F. Pearson correlation was used in Figures 1, 2, 3, 7, and Figures S2C and S3E.

### Code and Data Availability

The raw sequence data of RNA‐seq, CUT&Tag and ATAC‐seq generated during endosperm development in this study was deposited in the Genome Sequence Archive (https://bigd.big.ac.cn/gsa) under accession number PRJCA022666. The embryo sac data was downloaded from a previous study under accession number PRJCA008382.^[^ ^45^ ^]^

The data analysis method and code were based on a previous study at github (https://github.com/LongZhao1992/Dynamic‐chromatin‐regulatory‐programs‐during‐embryogenesis‐of‐hexaploid‐wheat),^[^ ^45^ ^]^ and specific data or code are available upon request.

## Conflict of Interest

The authors declare no conflict of interest.

## Author Contributions

L.Z., J.C., and Z.Z. contributed equally to this work. J.X. designed and supervised the research. J.X., D.‐Z.W., L.Z., Z.‐H.Z., and J.‐C.C. wrote the manuscript. L.Z., Z.‐H.Z., and D.‐Z.W. performed bioinformatics analysis. L.Z. and L.‐X.L. did ATAC‐seq and Cut&Tag experiments. W.‐Y.W, D.‐Z.W., and D.‐C.L. did phenotyping and RNA‐seq for 102 wheat accessions. D.‐Z.W. and P.Z. did the haploid and selection analysis. J.‐C.C. and M.‐X.G. did *Taabi3* mutants phenotype and transcription regulation assay. X.‐L.L. did in situ hybridization. C.‐F.Y. measured the protein content of *Taabi3* knock‐out transgenic lines. M.‐X.G. and D.‐C.L. provide *Taabi3* knock‐out transgenic lines. X.‐L.L., D.‐C.L., Y.‐Y.Y., and A.‐M.Z. revised the manuscript. D.‐Z.W., L.Z., Z.‐H.Z., J.‐C.C., Y.‐M.Y., and J.X. prepared all the figures. All authors discussed the results and commented on the manuscript.

## Supporting information

Supporting Information

Supplemental Tables 1‐13

## Data Availability

The data that support the findings of this study are available in the supplementary material of this article.
