# Supplementary material for: Deciphering the Transcriptional Regulatory Network Governing Starch and Storage Protein Biosynthesis in Wheat for Breeding Improvement
Source: Adv Sci (Weinh). 2024 Jun 28;11(33):2401383. doi: 10.1002/advs.202401383 (PMC11434112; doi:10.1002/advs.202401383)
Supplement: Supplementary file 1 — Supporting Information [file ADVS-11-2401383-s002.docx]

**Supplemental figures and legends**

**
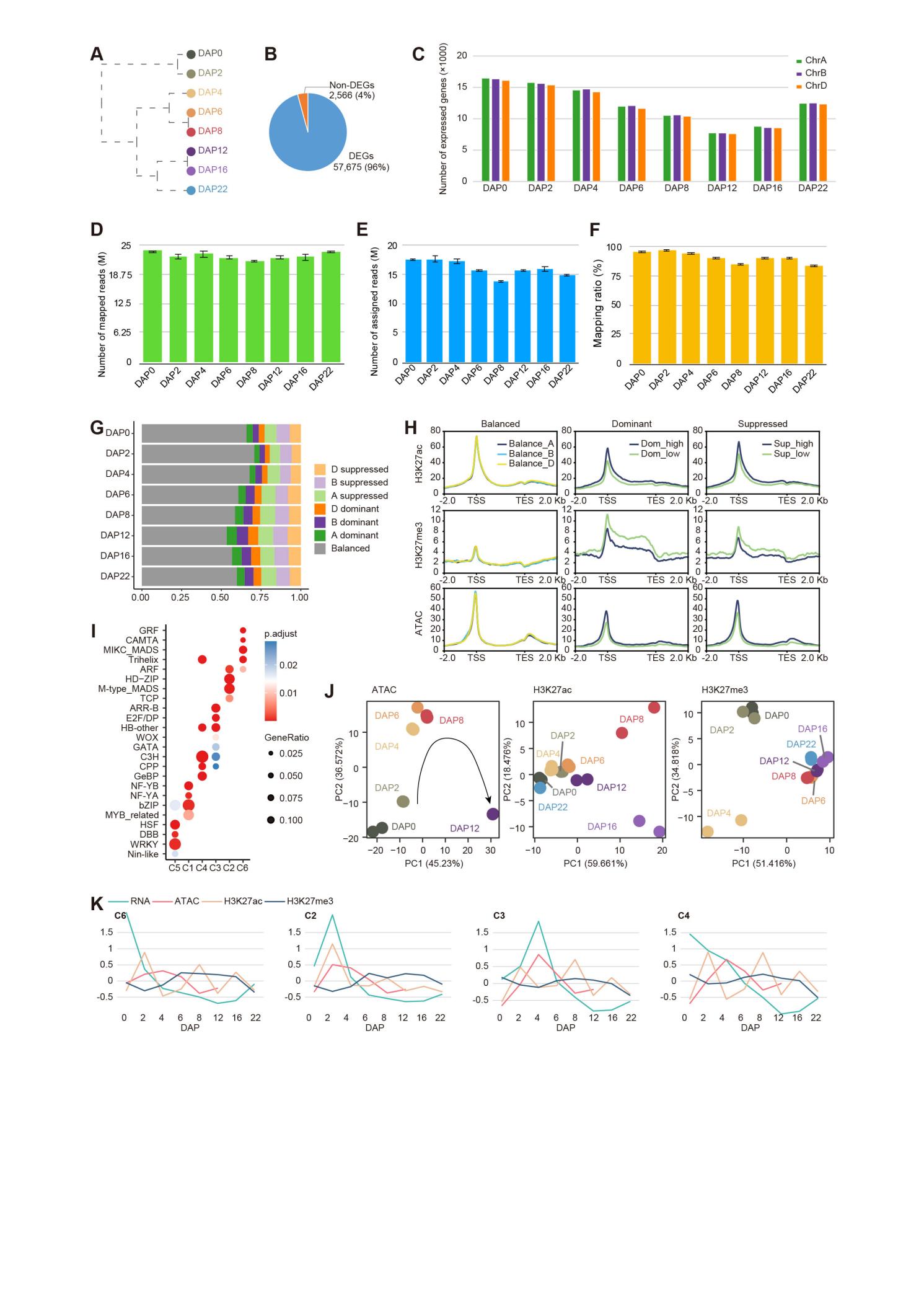
**

**Figure S1. Overview of transcriptome and epigenetic modification during endosperm development in wheat**

**A**. Cluster dendrogram of RNA-seq data for DAP0-22.

**B**. Proportion of DEGs to total expressed genes in endosperm. The DEGs was calculated between every two samples.

**C**. The number of expressed genes in A, B, D sub-genomes at different stages.

**D-F**. The number of mapped reads (reads assigned to the genome) (D), number of assigned reads (reads under coding region of the genome) (E), and mapping ratio (F) of different samples.

**G**. Proportion of different subgenome bias expression cluster genes at each sampling stage.

**H**. Mega profile of epigenetics modification on bias expression genes. “Dominant high” and “Suppressed high” represent the high expressed allele genes of one triad, while “Dominant low” and “Suppressed low” represents the low expressed allele genes of one triad.

**I**. TFs family’s enrichment from different cluster genes.

**J**. PCA analysis of ATAC, H3K27ac and H3K27me3.

**K**. Correlation between genes’ expression and epigenetics modification in different clusters. Line plot showing the dynamic of Z-score normalized average value of gene expression TPM and epigenetic modification peaks CPM at different DAP.

**
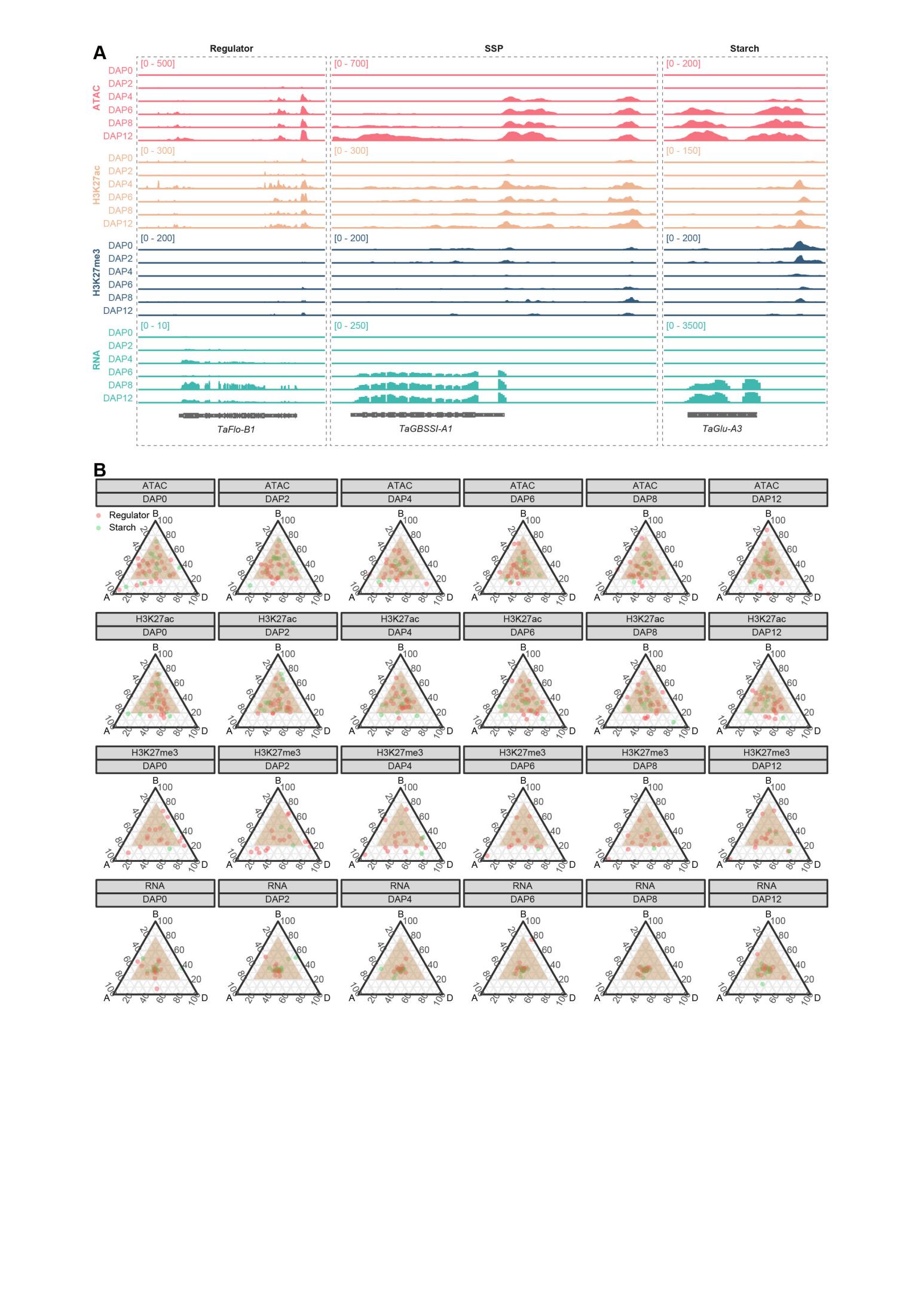
**

**
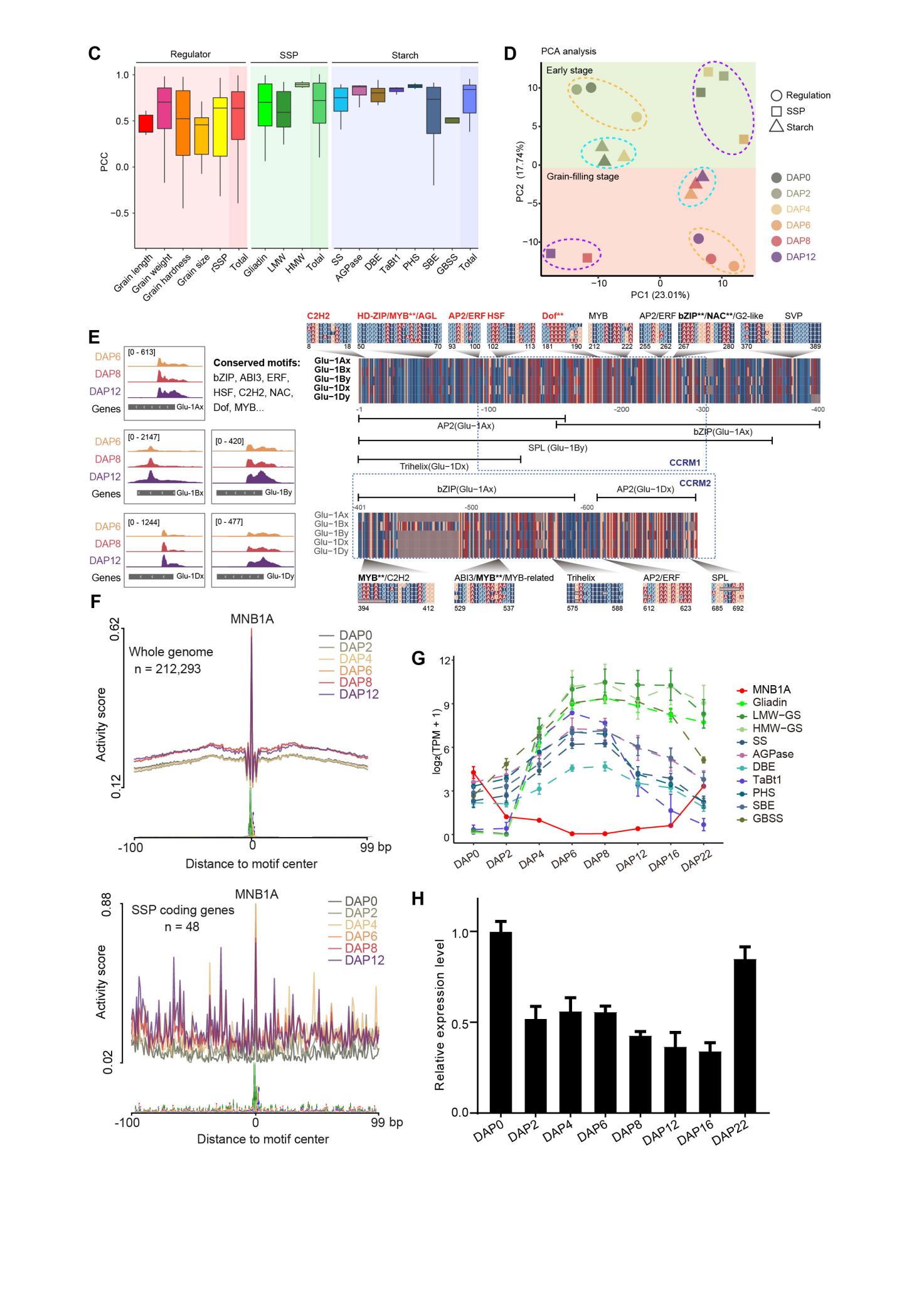
**

**Figure S2. Association of epigenetic modification with starch and SSP coding genes**

A. Chromatin accessibility, H3K27ac and H3K27me3 profile at representative genes from Starch, SSP, and Regulator-genes.

B. Relative expression and epigenetics modification (ATAC, H3K27ac and H3K27me3) abundance of starch synthesis and regulators genes during endosperm development. Each circle represents a gene triad with an A, B, and D coordinate consisting of the relative contribution of each homoeolog to the overall triad. Balanced triads are shown within brown shadow.

C. The boxplot of pearson correlation coefficient (PCC) between genes expression across all subgroups and chromatin accessibility at proximal accessible regions (pACRs). PCC values are shown separately for each subgroup and the entire group.

D. The PCA analysis of variation in TF binding motif activity based on chromVAR.

E. IGV screenshot showing the similar patterns of ATAC-seq signal around HMW-GS, and TF binding motifs in the promoters of HMW-GS genes.

F. TF footprints derived from ATAC-seq reads over NMNB1A motifs within accessible ATAC-seq regions at the whole-genome level (upper panel) or in SSP coding genes (lower panel) during endosperm development, with 'n' indicating the number of loci be hit; activity score reflects the chromatin accessibility.

1. Gene expression patterns of MNB1A, SSP coding genes and starch synthesis genes, and the error bars represent the average value ± standard error (SE).
2. The relative expression level of MNB1A across endosperm developmental stages. qRT-PCR data were normalized to *TaActin*, with values from three replicates shown as mean ± sd.


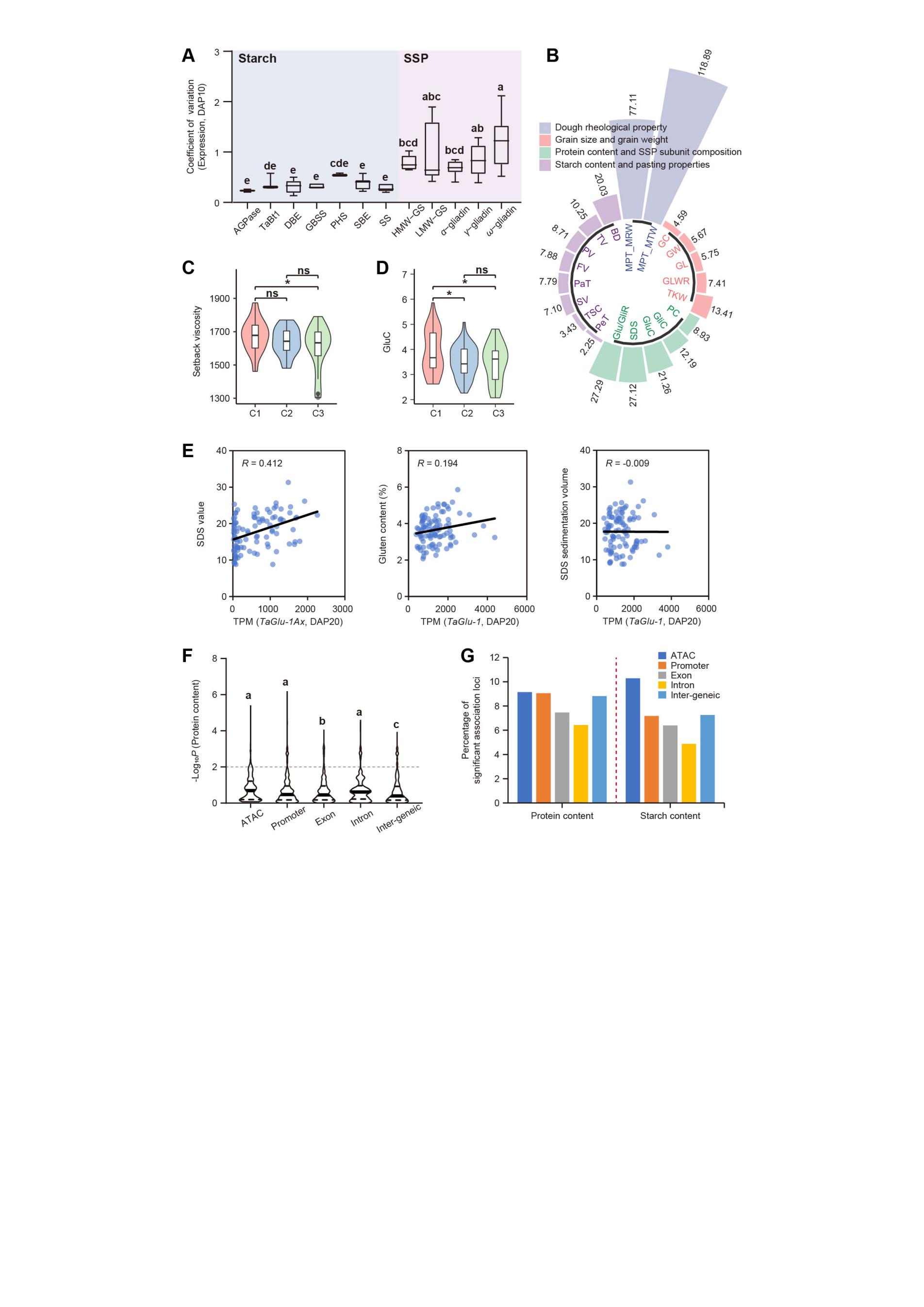


**Figure S3. Expression diversity of starch and SSP-related genes within wheat population**

1. The comparison of coefficient of variation (CV) in expression levels for each subtype genes within the core collection of 102 wheat accession at DAP10. The box denotes the 25^th^, median and 75^th^ percentiles, and whiskers indicate 1.5 × interquartile range. The LSD multiple comparisons test was used to determine the significance of CV differences among subtype genes. Different letters indicate a significant difference at *P* ≤ 0.05.
2. Rose plots indicating the CV of each trait across 102 wheat accessions.
3. Comparison of the setback viscosity (SV) of wheat varieties among three groups clustered by *TaBt1* expression level. The student’s t-test was used to determine the statistical significance between two groups. *, *p* ≤ 0.05; ns, no significant difference.
4. Comparison of the gluten contents of wheat varieties among three groups clustered by *TaGlu-1Ax* expression level. The student’s t-test was used to determine the statistical significance between two groups. *, *p* ≤ 0.05; ns, no significant difference.
5. Scatter plot of gluten content (left panel) and SDS sedimentation volume (middle panel) against the total expression level of *TaGlu-1*, and SDS sedimentation volume against the expression level values of *TaGlu-1Ax* (right panel) in corresponding accessions at DAP20. Each dot denotes one accession, and the black line represents the regression trend calculated by the general linear model.
6. Comparison of the -log_10_(*p* value) of SNPs with storage protein content among different genomic feature. The LSD multiple comparisons test was used to determine the significance differences among subtype genes. Different letters indicate a significant difference at *P* ≤ 0.05.
7. The percentage of significant association loci with storage protein content and starch content among different genomic feature. The SNP with -log_10_(*p* value) ≥ 2 were considered as significant association loci.


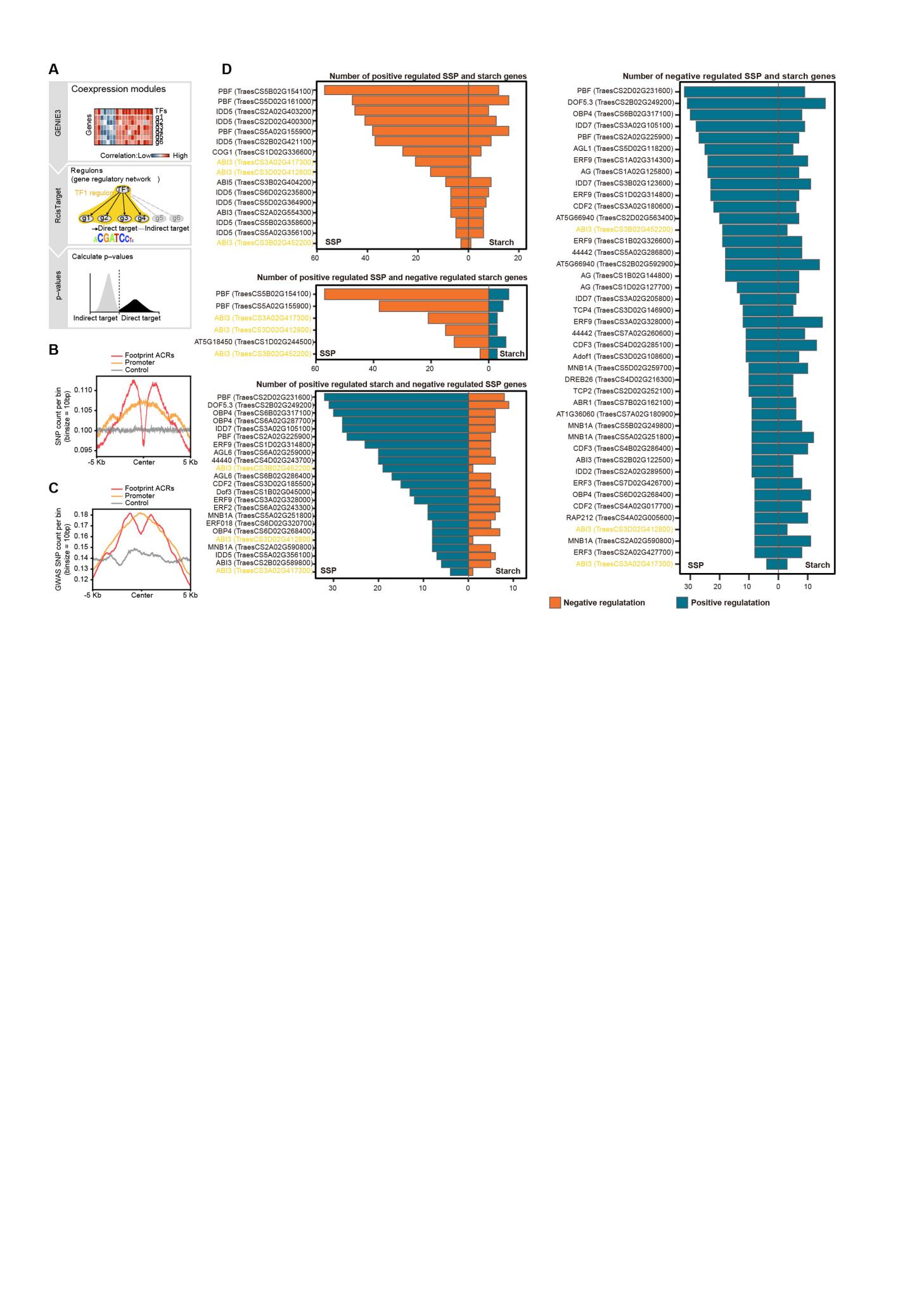


Figure S4. Transcriptional regulatory networks (TRNs) construction

A. The flow-chart showing the strategy used for construction of TRNs.

B, C. The SNPs (B) and GWAS SNPs (C) distribution around accessible chromatin regions (ACRs) of targets. The footprint ACRs represent the open chromatin regions at the promoter of target genes and can be bound by the TFs. Control represented the random select regions from whole genome.

**D**. The number of positively and negatively regulated starch and SSP related genes for specific TFs. Color bar: brown, positive regulation; blue, negative regulation.


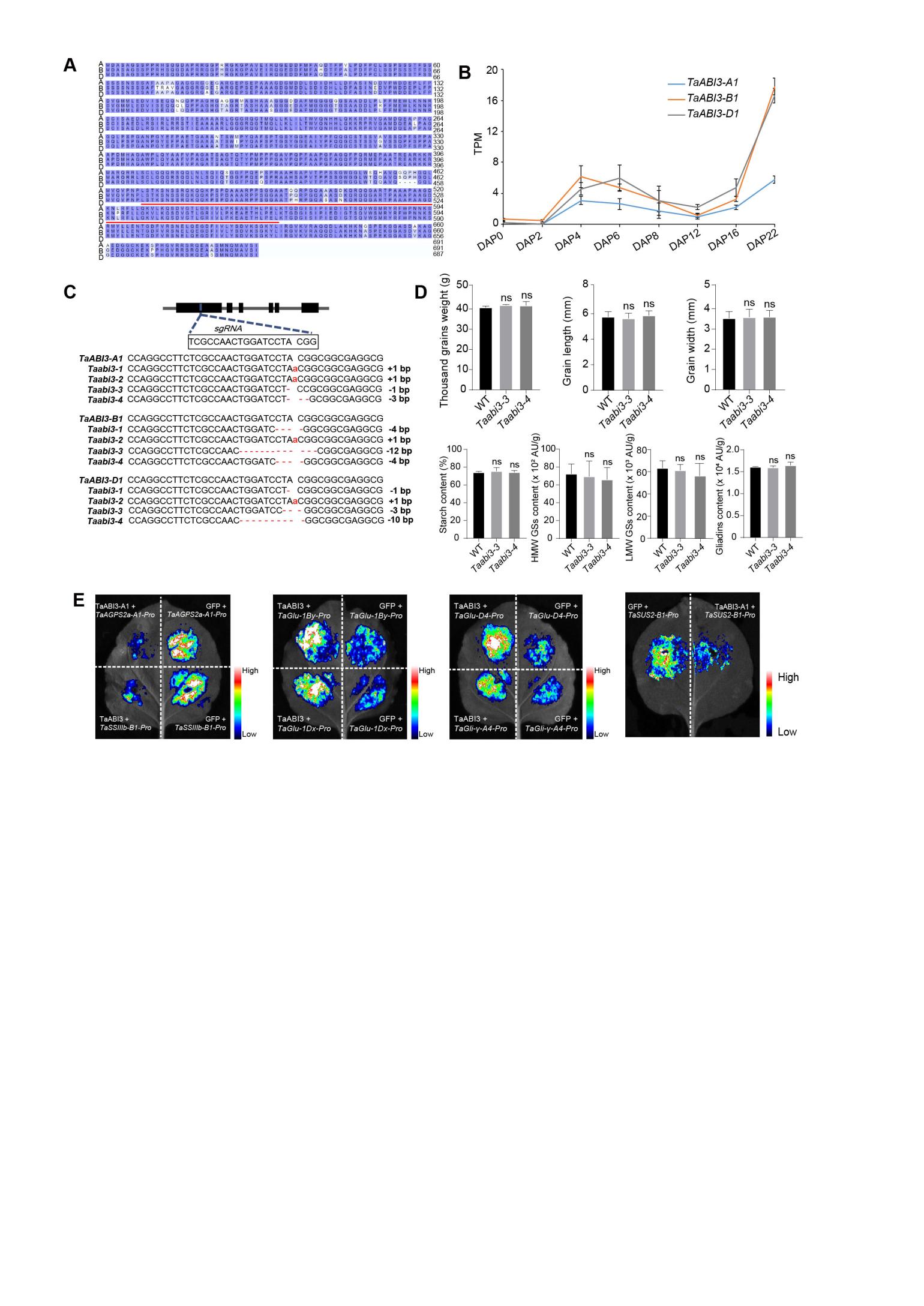


Figure S5. Generation of *Taabi3* mutant lines

**A**. Protein sequence alignment of *TaABI3-A1* and its homologous genes. The conserved B3 domain is denoted by a red line.

B. The expression dynamic of *TaABI3-A1* and its homologous genes across the endosperm developmental stages.

C. Schematic diagram showing the construction of *Taabi3-KO* lines.

D. Comparison of the grain size related traits, content of starch and SSP components between wild-type and *Taabi3-3* and *Taabi3-4* mutant lines. Three repeats were carried out for each sample to measure the amounts of starch, HMW-GSs, LMW-GSs, and gliadins content. Student’s t-test was used to determine the difference significance between *Taabi3* mutant and wild-type. ns, no significant difference.

E. Representative images for the LUC reporter assay used to evaluate the transcriptional regulation of TaABI3-A1 to its targets involved in starch and SSP biosynthesis.

Supplemental Tables

Supplementary Table S1. The TPM values of expressed genes during endosperm development

Supplementary Table S2. Clustering of DEGs among developmental stages

Supplementary Table S3. The gene expression of SSP coding genes, starch synthesis genes and regulators during endosperm development

Supplementary Table S4. The expression level and coefficient of variation of SSP-set, Starch-set genes in wheat population

Supplementary Table S5. Correlation between the expression level of SSP-set, Starch-set genes and phenotypic traits in wheat population

Supplementary Table S6. Gene information of 436 core TFs from TRN

Supplementary Table S7. Grain size and quality GWAS data used in this study

Supplementary Table S8. The evaluation of 395 novel TFs

Supplementary Table S9. Correlation between the expression level of 395 novel genes and grain size and quality traits in wheat population

Supplementary Table S10. The KN9204 TILLING mutants for 395 novel genes

Supplementary Table S11. The expression, TRN and mutant information for the 42 key TFs.

Supplementary Table S12. TaABI3-A1 haplotype of MCC population
Supplementary Table S13. Primers used in this study
